# Phytochemical Investigation of *Gyrinops vidalii* Leaves and Evaluation of the *α*-Glucosidase Inhibitory Activity of the Isolated Metabolites and Molecular Docking of the Active Compounds

**DOI:** 10.3390/plants15162474

**Published:** 2026-08-15

**Authors:** Siriwan Srisit, Thittaya Ketnad, Khemika Singmahan, Pasakorn Bunchalee, Worrawat Promden, Panawan Moosophon, Vanida Choomuenwai, Bunleu Sungthong, Nadtanet Nanthaboot, Anake Kijjoa, Prapairat Seephonkai

**Affiliations:** 1 Department of Chemistry and Center of Excellence for Innovation in Chemistry (PERCH-CIC), Faculty of Science, Mahasarakham University, Maha Sarakham 44150, Thailand; srisitsiriwan@gmail.com (S.S.); tittayakatnad@gmail.com (T.K.); khemika15728@gmail.com (K.S.); nadtanet@gmail.com (N.N.); 2 Department of Biology, Faculty of Science, Mahasarakham University, Maha Sarakham 14450, Thailand; pasakorn.b@msu.ac.th; 3 Division of General Science, Faculty of Education, Buriram Rajabhat University, Buriram 31000, Thailand; worrawat.pd@bru.ac.th; 4 Department of Chemistry and Center of Excellence for Innovation in Chemistry, Khon Kaen University, Khon Kaen 40002, Thailand; mpanaw@kku.ac.th; 5 Department of Chemistry, Faculty of Science and Technology, Nakhon Ratchasima Rajabhat University, Nakhon Ratchasima 30000, Thailand; vanida.c@nrru.ac.th; 6 Faculty of Pharmacy, Mahasarakham University, Maha Sarakham 44150, Thailand; bunleu.s@msu.ac.th; 7 School of Medicine and Biomedical Sciences Abel Salazar (ICBAS) and CIIMAR, Universidade do Porto, 4050-313 Porto, Portugal

**Keywords:** *Gyrinops*, agarwood, mangiferin, benzophenone glycosides, iriflophenone, blumenol A, loliolide, *α*-glucosidase inhibitory activity

## Abstract

*Gyrinops vidalii* (Thymelaeaceae), a critically endangered agarwood-producing species native to Thailand and Laos and remains phytochemically unexplored. In this study, the chemical constituents and biological activities of the ethyl acetate (EtOAc) fraction of methanol extract of *G. vidalii* leaves were investigated. Phytochemical analysis led the isolation of mangiferin (**1**), iriflophenone 3-*C*-*β*-D-glucoside (**2**), aquilarinenside E (**3**), iriflophenone 2-*O*-*α*-L-rhamnoside (**4**), 5,7,4′-trimethoxyflavone (**5**), blumenol A (**6**), loliolide (**7**), 4-hydroxybenzoic acid methyl ester (**8**), and 4-hydroxybenzoic acid (**9**). Compound **1** exhibited potent DPPH radical scavenging activity with an SC_50_ value of 19.35 μM), whereas iriflophenone (**3a**), obtained by acid hydrolysis of **3**, and **5** showed promising *α*-glucosidase inhibitory activity with IC_50_ values 100.06 and 174.57 μM, respectively. Molecular docking demonstrated favorable binding of both compounds within the *α*-glucosidase active site through hydrogen bonding, aromatic, and hydrophobic interactions, with binding energies of −7.4 and −8.0 kcal/mol, respectively. Predicted ADMET properties further supported the drug-like potential of both compounds, indicating favorable aqueous solubility, high intestinal absorption, and no predicted hepatotoxicity, while differences were observed in their predicted blood–brain barrier permeability. This study represents the first phytochemical investigation of *G. vidalii*, provides scientific support for its traditional use, and expands the chemotaxonomic knowledge of the closely related genera *Gyrinops* and *Aquilaria*.

## 1. Introduction

*Aquilaria* and its closely related genus, *Gyrinops*, are recognized as sister genera within the family Thymelaeaceae (tribe Aquilarieae, order Malvales) [1,2]. Both genera are well known as major producers of agarwood, a highly valued fragrant agarwood type resin known as gaharu, oud, or lign-aloes. Agarwood is formed as a defense response to fungal infection or physical injury and has been extensively utilized in traditional medicine, religious ceremonies, perfumery, and aromatherapy across Asia and the Middle East [3,4,5,6]. To date, 21 species of *Aquilaria* and 9 species of *Gyrinops* have been recorded [7]. The currently recognized *Gyrinops* species include *G. caudata*, *G. decipiens*, *G. ledermannii*, *G. moluccana*, *G. podocarpus*, *G. salicifolia*, *G. versteegii* (Indonesia and Papua New Guinea), *G. walla* (Indonesia, Papua New Guinea, India, and Sri Lanka), and *G. vidalii* (Thailand and Laos) have been recorded [2,8,9].

Among the *Gyrinops* species, *G. vidalii* P.H.Hô (Thai name: “Kritsana Noi”) is the only species native to Thailand and Laos. This species was first recorded in Nong Khai Province, northeastern Thailand [10], and is currently classified as critically endangered species due to its restricted distribution and overexploitation for agarwood production [11]. In Laotian traditional medicine, the leaves of *G. vidalii* are used for the treatment of diabetes and allergies, and its wood is believed to promote blood circulation and improve overall health [12].

Phytochemical studies of *Gyrinops* species are limited. Previous investigations have reported triterpenoids and 2-(2-phenylethyl)chromones from the leaves and the agarwood [13,14], and volatile compounds from the resin [15] of *G. walla*, and sesquiterpenoids and 2-(2-phenylethyl)chromones from agarwood of *G. salicifolia* [16], some of which exhibited acetylcholinesterase inhibitory and moderate cytotoxicity against K562, BEL-7402, and SGC-7901 human tumor cell lines [16,17,18]. However, no phytochemical study of *G. vidalii* has been reported to date.

As part of our ongoing search for bioactive secondary metabolites from medicinal plants of northeastern Thailand, we investigated the ethyl acetate (EtOAc) fraction obtained from the methanolic leaf extract of *G. vidalii*, collected in Phuwua Wildlife Sanctuary, Bueng Kan Province, northeastern Thailand. To the best of our knowledge, this study represents the first phytochemical investigation of this native endangered species and provides valuable insights for its sustainable utilization.

## 2. Results and Discussion

### 2.1. Screening of Antioxidant Activity and Chemical Compounds

The crude methanol (MeOH) extract was first partitioned with hexane and EtOAc to obtain the hexane and EtOAc fractions. The MeOH extract, hexane and EtOAc fractions were then evaluated for 2,2-diphenyl-1-picrylhydrazyl (DPPH) radical scavenging activity, total phenolic content (TPC), and total flavonoid content (TFC). The results showed that the EtOAc fraction exhibited the strongest DPPH radical scavenging activity and the highest TPC and TFC values (Table 1). The ^1^H NMR spectrum (Figure S2) of this fraction (recorded in CD_3_OD) displayed characteristic signals corresponding to aromatic protons and sugar moieties of secondary metabolites. Therefore, the EtOAc fraction was selected for further phytochemical investigation.

### 2.2. Phytochemical Investigation

Extraction of the dried leaves of *G. vidalii* with MeOH afforded a MeOH layer, from which mangiferin (**1**) precipitated and was collected by filtration. Additionally, precipitated of **1** was also collected from the EtOAc layer obtained from solvent partitioning. Compounds **2**–**7** were obtained through column chromatographic separation. The structures of the isolated compounds were elucidated based on analysis of their nuclear magnetic resonance (NMR) spectroscopic data, high-resolution electrospray ionization mass spectrometry (HRESIMS), mass spectrometry (MS), as well as comparison of their NMR data with those reported in the literature.

**Figure 1:**
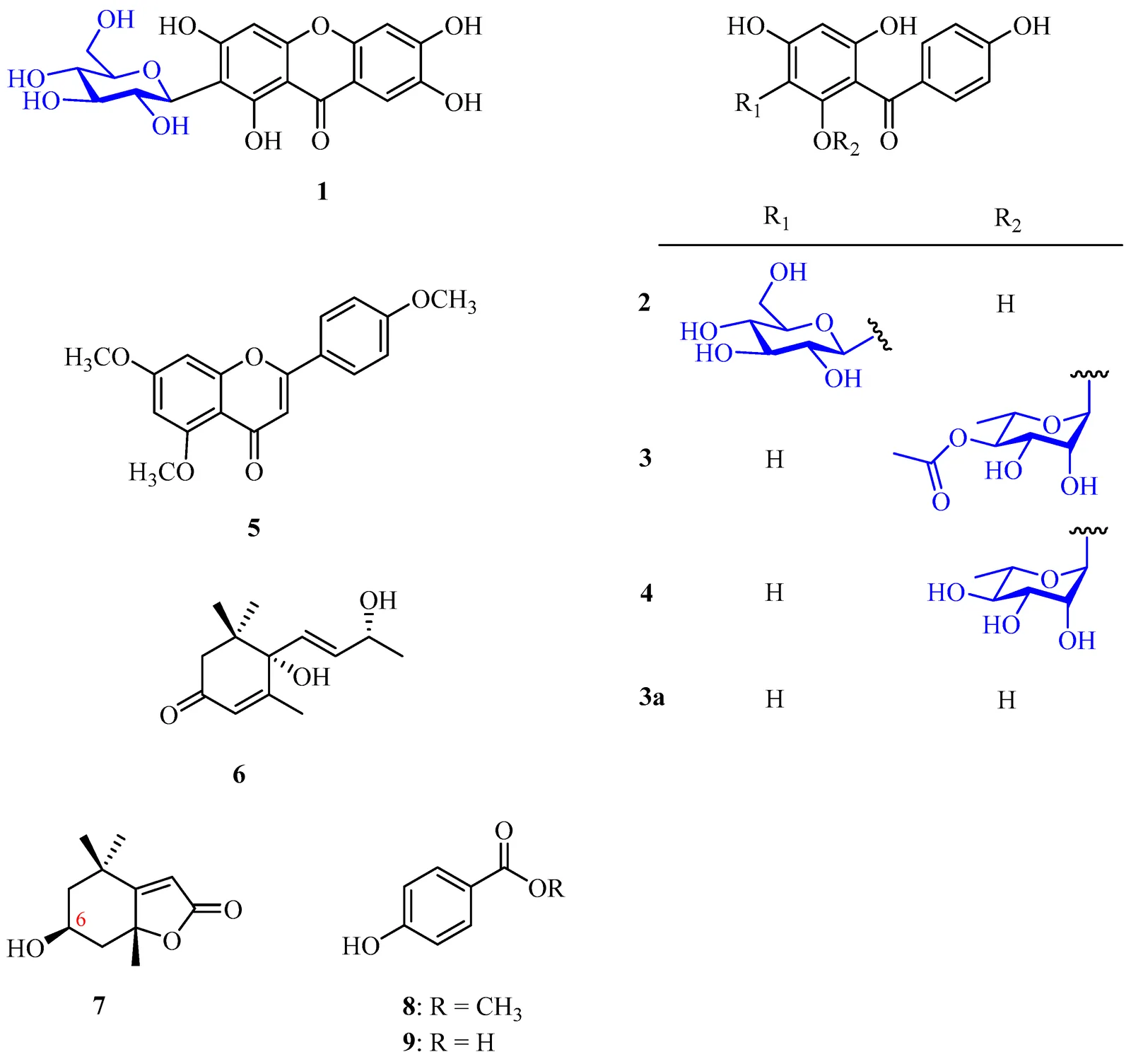
Structures of compounds **1**–**9** and **3a**.

The isolated compounds (**1**–**7**) were identified as mangiferin (**1**) [19,20], iriflophenone 3-*C*-*β*-D-glucoside (**2**) [21], aquilarinenside E (**3**) [22], iriflophenone 2-*O*-*α*-L-rhamnoside (**4**) [23,24], 5,7,4′-trimethoxyflavone (**5**) [25], blumenol A (vomifoliol) (**6**) [26,27], loliolide (**7**) [28,29], 4-hydroxybenzoic acid methyl ester (**8**) [30], and 4-hydroxybenzoic acid (**9**) [31] (Figure 1). The isolated compounds **1**–**7** have previously been reported from species within the genus *Aquilaria*.

Compound **1**, originally isolated from *Mangifera indica* (Anacardiaceae), is widely distributed in higher plants but is particularly abundant in the stem bark, leaves, peels, and kernels of *M. indica*, with reported yields of up to 7.1% in the stem bark [32,33,34]. Owing to its broad range of pharmacological activities, mangiferin has attracted considerable interest for pharmaceutical and functional food applications [33,35,36,37,38]. However, its highly polar nature, resulting from a glucose moiety and multiple hydroxyl groups, presents challenges for its extraction, isolation, and purification despite its good solubility in polar organic solvents such as methanol and ethanol [35].

The isolation of **1** from the leaves of *Aquilaria sinensis* has previously been reported [39,40,41], as well as from *A. crassna* [42,43] and *A. walla* [44]. Compound **1** together with the polyphenolic constituent genkwanin 5-*O*-*β*-primeveroside, has been identified as one of the two major bioactive constituents in *A. sinensis* leaf extract [45]. In the present study, compound **1** was isolated from the dried leaves of *G. vidalii* with an approximate yield of 1.78%. Because of its high polarity and high abundance in the leaves, it precipitated quantitatively from the concentrated MeOH extract solution, with additional amounts subsequently precipitating from the EtOAc-soluble fraction.

Benzophenone glycosides represent one of the major classes of bioactive constituents identified in the leaves, stems, and roots of *Aquilaria* species. The occurrence of iriflophenone glycosides, including compound **2** from the leaves of *A. sinensis* [39,40,41], **3** from the leaves and flower buds of *A. sinensis* [22,46] and the pericarps of *A. yunnanensis* [24], and **4** from the agarwood leaves and stems of *A. sinensis* [47,48] as well as the leaves of *A. crassna* [42], has previously been reported. In the present study, compound **4** (iriflophenone 2-*O*-*α*-L-rhamnoside) was isolated as the second major constituent, with an approximate yield of 0.22% from the dried leaves of *G. vidalii*.

The present findings provide additional chemotaxonomic evidence supporting the close relationship between *Gyrinops* and *Aquilaria*. This is consistent with recent phylogenomic studies based on complete plastome sequences, which revealed a close phylogenetic affinity between the two genera and suggested that their current taxonomic delimitation may require re-evaluation [2]. Recently, molecular and morphological analyses of *A. khasiana* revealed that *Gyrinops* is paraphyletic to *Aquilaria* [49].

Acid hydrolysis of compound **3** yielded its aglycone, **3a**, which was iriflophenone. The structure of **3a** was confirmed by analysis of its ^1^H NMR spectroscopic data and comparison with literature values [50]. Iriflophenone (**3a**) has previously been reported from the leaves of *A. sinensis* [39]; however, its occurrence as a free compound has not yet been documented in *Gyrinops* species. Outside the family Thymelaeaceae, iriflophenone has also been isolated from the underground parts of *Iris potaninii* [51] and *I. florentina* [52].

Flavonoids and other phenolic compounds are widely distributed throughout the plant kingdom and play important roles in the human diet due to their antioxidant properties. However, only a few isoflavones have been reported from *A. sinensis*. Among them, compound **5** was previously isolated from the leaves of *A. sinensis* together with tirucallane-type triterpenoids [53].

Sesquiterpenes and chromones are recognized as the principal constituents of agarwood. Among them, sesquiterpenoids are the major chemical components responsible for its characteristic fragrance and various medicinal properties [54,55,56]. Numerous structurally diverse sesquiterpenes have been reported from *A. sinensis*, *A. malaccensis*, *A. crassna*, and *A. subintegra* [57]. In contrast, only a limited number of sesquiterpenoids have been documented from *Gyrinops*, with reports restricted to *G. salicifolia* [17]. In the present study, a degraded sesquiterpene, blumenol A (**6**), and a monoterpene lactone loliolide (**7**) were isolated. Compound **6** has previously been reported from the stems of *A. sinensis* [58], whereas compound **7** was detected in leaf extracts of *A. malaccensis* by gas chromatography–mass spectrometry (GC–MS) analysis [59].

Recently, compound **7** (6*S*-loliolide) (Figure 1) and its epimer, *epi*-loliolide (6*R*), were isolated from the crude dichloromethane extract of the microalga *Thalassiosira* sp., and their structures were unambiguously established by 2D NMR spectroscopy [29]. The absolute configuration of **7** was further confirmed by X-ray crystallography analysis [29]. The ^1^H and ^13^C NMR spectroscopic data of **7** and its epimers, recorded in CDCl_3_, exhibit characteristic differences at the stereogenic C-6 position. For **7**, H-6 and C-6 appeared at *δ*_H_ 4.34, (m) and *δ*_C_ 66.7, whereas the corresponding signals for *epi*-loliolide appeared at *δ*_H_ 4.10 (m) and *δ*_C_ 64.8. The structure of **7** was assigned by comparison of its ^1^H and ^13^C NMR spectroscopic data recorded in CDCl_3_ with those reported in the literature, particularly the chemical shifts in H-6 (*δ*_H_ 4.33 ppm, m) and C-6 (*δ*_C_ 66.8 ppm), which are in excellent agreement with the reported values for *6S*-loliolide (Table S7).

Compound **7** has been previously reported from a few macroalgal species [60,61,62], a marine microalga [63], and terrestrial plant species [64,65,66]. Given its occurrence in phylogenetically diverse terrestrial plants and marine algae, the biosynthetic origin of **7** remains unclear, and the possible contribution of endophytic microorganisms to its production should be further investigated.

### 2.3. DPPH Radical Scavenging and α-Glucosidase Inhibitory Activities

The DPPH radical scavenging and *α*-glucosidase inhibitory activities of compounds **1**–**5** and **3a** were evaluated because the EtOAc fraction exhibited notable radical scavenging activity during preliminary screening and the leaves of *G. vidalii* are used for the treatment of diabetes in Laotian traditional medicine [12]. Based on the preliminary screening results (Table S12), only compounds showing appreciable activity were selected for further evaluation of their SC_50_ (50% scavenging concentration) and IC_50_ (50% inhibitory concentration) values. Due to the limited quantities of compounds **6** and **7** obtained from the isolation process, as well as the common occurrence of phenolic acid derivatives **8** and **9** in plants, these compounds were not subjected to biological evaluation.

Among the tested compounds, only compound **1** exhibited notable DPPH radical scavenging activity, with an SC_50_ value of 19.35 μM (Table 2). This activity was ca. 2-fold stronger than that of ascorbic acid (SC_50_ = 41.90 μM), which was used as the positive control. Compound **1** has been extensively investigated for its broad spectrum of therapeutic effects, including antioxidant activity, which is well established [33,35,36,37,38]. The occurrence of **1**, which exhibits diverse health-promoting activities, provides support for the traditional use of *G. vidalii* leaves as a medicinal plant.

Although compounds **3a** and **2**–**4** contain phenolic hydroxy groups in their structures, they did not exhibit DPPH radical scavenging activity. This observation is consistent with the findings reported by Malherbe et al. [67]. Flavonoids are known to play an important role in antioxidant activity due to the presence of free phenolic hydroxy groups, which can donate hydrogen atoms to neutralize free radicals. Therefore, the number and position of phenolic hydroxy groups are important determinants of antioxidant capacity. Replacement of hydroxy groups with methoxy groups generally reduces antioxidant activity. Accordingly, compound **5** (5,7,4′-trimethoxyflavone), which contains only methoxy substituents and lacks free phenolic hydroxy groups, did not exhibit antioxidant activity.

For *α*-glucosidase inhibitory activity, **5** and **3a** exhibited promising effects, with IC_50_ values of 174.57 and 100.06 μM, respectively (Table 2). These activities were ca. 1.4- and 2.4-fold more potent than that of acarbose (IC_50_ = 237.13 μM), which served as the positive control in this assay.

Benzophenone **3a** (iriflophenone) exhibited a stronger *α*-glucosidase inhibitory activity than acarbose. Previous studies on hydroxybenzophenones have demonstrated that increasing the number of phenolic hydroxyl groups on the benzophenone scaffold enhances *α*-glucosidase inhibitory activity [68]. To date, several benzophenones have been isolated from plants and microorganisms, and the biological activities of simple oxygenated benzophenones and their glycosides have recently been reviewed [69]. In particular, anti-inflammatory activity was reported for **2** and **3** [70], whereas antibacterial activity has been described for **2** [71]. Based on various biological activities of benzophenones, chemical structure modification could be performed to enhance biological activities.

Owing to the promising *α*-glucosidase inhibitory activity of **3a** and **5**, their physicochemical and absorption, distribution, metabolism, excretion, and toxicity (ADMET) calculations and molecular interactions with *α*-glucosidase were further investigated.

### 2.4. Computational Study

#### 2.4.1. Molecular Docking Study

Before docking compounds **3a** and **5**, the docking protocol was evaluated by docking maltose, the co-crystallized ligand of *S. cerevisiae* isomaltase, into the active site of the validated homology model using the same docking parameters applied throughout this study. Since the experimentally determined three-dimensional structure of the target *α*-glucosidase is not available, the homology model was employed for all docking calculations. The predicted binding pose of maltose showed an RMSD of approximately 3.0 Å relative to the crystallographic pose in the template structure (PDB ID: 3A4A) (Figure S1). Although RMSD values below 2.0 Å are generally considered indicative of excellent agreement, values below 4.0 Å are regarded as acceptable for reproducing experimental binding modes [72]. Therefore, the obtained RMSD supports the suitability of the docking protocol for the subsequent docking analyses.

**Figure 2:**
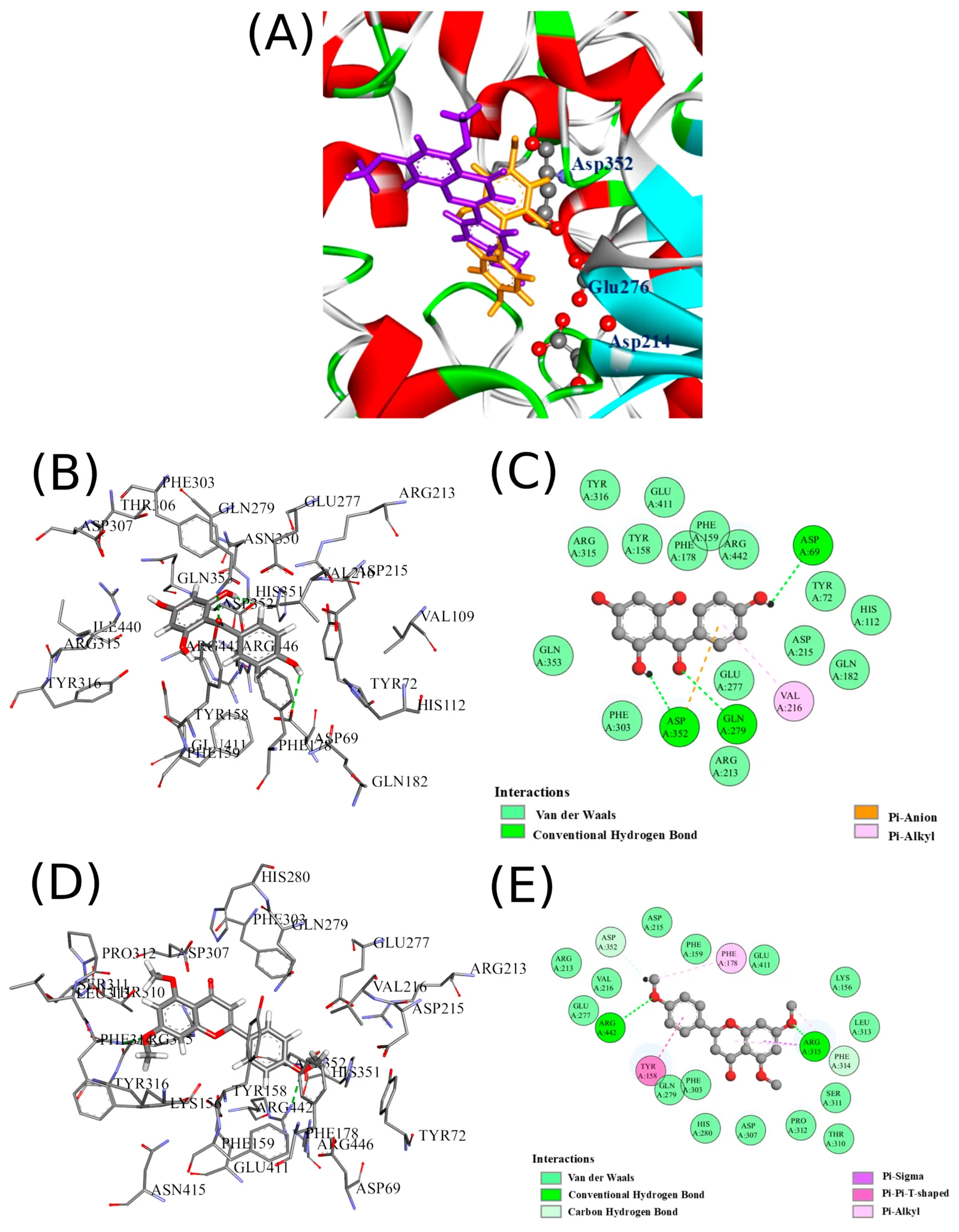
(**A**) Overlay of the docked poses of **3a** (orange) and **5** (purple) in the *α*-glucosidase binding pocket. Catalytic residues are shown as ball-and-stick models. (**B**,**C**) 3D and 2D interaction diagrams of **3a**. (**D**,**E**) 3D and 2D interaction diagrams of **5**.

Binding modes of compounds **3a** and **5** within the *α*-glucosidase binding pocket are illustrated in Figure 2. Both ligands were located close to the key catalytic residues Asp215, Glu277, and Asp352 (residue numbers based on 3A4A), which correspond to Asp214, Glu276, and Asp349 in the original *α*-glucosidase protein, the numbering difference arising from the residue indexing used during receptor preparation. Their binding positions were also comparable to that of maltose, a well-established competitive inhibitor [73].

The predicted binding energies of **3a** and **5** were −7.4 and −8.0 kcal/mol, respectively. Although compound **5** showed a slightly more favorable predicted binding energy than **3a**, the difference in predicted binding energies is relatively small, suggesting that both compounds may exhibit comparable binding affinities toward *α*-glucosidase. Compound **3a** was stabilized by a combination of hydrogen bonding, hydrophobic contacts, and aromatic interactions. Three conventional hydrogen bonds were formed with Asp69, Gln279, and Asp352, while hydrophobic interactions involved several aromatic residues, including Tyr72, Tyr158, Phe159, Phe178, and Phe303. In addition, the aromatic core of the ligand established π-anion and π-alkyl interactions with Asp352 and Val216, respectively, further supporting its binding within the active-site pocket. In contrast, **5** formed two conventional hydrogen bonds with Arg315 and Arg442, together with numerous van der Waals contacts involving Phe159, Val216, Phe303, Pro312, Phe314, and Leu313. Although **5** formed fewer hydrogen bonds than **3a**, it exhibited a more extensive network of aromatic interactions, including π–π T-shaped interactions with Tyr158 and Phe178, as well as additional π-alkyl interactions with Arg315. These interactions likely compensate for the smaller number of hydrogen bonds and may account for the slightly more favorable binding energy of **5**.

Overall, both compounds occupied a similar region of the catalytic pocket but adopted distinct interaction patterns. Compound **3a** relied primarily on hydrogen-bonding interactions, whereas **5** appeared to derive greater stabilization from aromatic and hydrophobic contacts. These differences suggest that the two ligands employ complementary binding strategies while maintaining comparable binding orientations within the α-glucosidase active site.

#### 2.4.2. Drug Likeness and ADMET Properties

Drug likeness and oral bioavailability of **3a** and **5** were evaluated using Lipinski’s Rule of Five (Ro5), which is commonly applied as an initial filter for identifying compounds with favorable oral drug properties. According to the Ro5 criteria, compounds are expected to exhibit acceptable oral bioavailability when they have a molecular weight (MW) < 500 Da, a LogP value < 5, no more than five hydrogen bond donors (HBDs), and no more than ten hydrogen bond acceptors (HBAs) [74]. As summarized in Table S10, both compounds met all Ro5 requirements. Compound **3a** has a molecular weight of 246.2 Da, a LogP value of 1.74, four HBDs, and five HBAs, while **5** has a molecular weight of 312.3 Da, a LogP value of 3.49, no HBDs, and five HBAs. Collectively, these findings indicate that both compounds possess physicochemical properties consistent with favorable oral drug likeness.

The predicted ADMET properties of **3a** and **5** are summarized in Table S11. Both compounds exhibited acceptable aqueous solubility (logS < −2) together with favorable human intestinal absorption, supporting their potential suitability for oral administration [75]. However, **5** showed a markedly higher predicted intestinal absorption (98%) than **3a** (61%). The predicted distribution profiles also differed between the two compounds. Compound **3a** exhibited a negative logBB value (−0.954), suggesting limited distribution across the blood–brain barrier, whereas **5** showed a positive logBB value (0.257), indicating a greater tendency to enter the brain. Despite this difference, both compounds had logPS values <−2, suggesting limited permeability into the central nervous system and, therefore, a low likelihood of extensive CNS exposure [76,77].

The predicted metabolic profiles revealed notable differences between **3a** and **5**. Neither of the two compounds was identified as a substrate or inhibitor of CYP2D6. Their CYP3A4 profiles, however, differed considerably: **3a** was identified as an inhibitor, whereas **5** was predicted to serve as a substrate. These findings suggest that **3a** and **5** may undergo different metabolic pathways and exhibit distinct potentials for CYP3A4-mediated drug–drug interactions. Differences were also observed in the predicted excretion profiles. Compound **5** exhibited a higher total clearance than **3a** (0.819 vs. 0.495 log mL/min/kg), consistent with a faster rate of elimination. In addition, only **5** was identified as a substrate of the renal organic cation transporter 2 (OCT2), suggesting that transporter-mediated renal excretion may contribute to its clearance. Both compounds were predicted to be non-mutagenic in the AMES assay and showed no evidence of hepatotoxicity. Taken together, these results indicate favorable pharmacokinetic and safety profiles for both compounds, supporting their further evaluation as potential orally active drug candidates. Nevertheless, since these results are based on computational models, they should be considered preliminary until confirmed by complementary in vitro and in vivo experiments.

## 3. Materials and Methods

### 3.1. General Experimental Procedures

NMR spectra were recorded on a Bruker Ascend-400 (Prodigy unit) (Bruker BioSpin, Rheinstetten, Germany) with deuterated solvents; CDCl_3_ (*δ*_H_ 7.26/*δ*_C_ 77.0 ppm), CD_3_OD (*δ*_H_ 3.31/*δ*_C_ 49.0 ppm), acetone-*d*_6_ (*δ*_H_ 2.05/*δ*_C_ 29.8), and DMSO-*d*_6_ (*δ*_H_ 2.50/*δ*_C_ 39.5 ppm) (Eurisotop, Cambridge Isotope Laboratories, Saint-Aubin, France). HRESIMS spectra were measured using a Bruker micrOTOF (Agilent Technologies, Santa Clara, CA, USA) mass spectrometer. UV absorbance values were recorded by UV/Vis spectrophotometer microplate reader (FLUOstar^®^ Omega, BMG LABTECH, Ortenberg, Germany). Column chromatography was performed using silica gel 60 (Merck, Darmstadt, Germany) and Sephadex LH-20 (Sigma Aldrich, St. Louis, MO, USA). Pre-coated silica gel 60 F_254_ on Aluminum sheets (Merck, Darmstadt, Germany) was used for analytical thin-layer chromatography (TLC).

### 3.2. Plant Material

The leaves of *Gyrinops vidalii* were collected in May 2023 from Phuwua Wildlife Sanctuary, Bueng Kan Province, northeastern Thailand, with permission from the National Park, Wildlife and Plant Conservation Department. The collection site is characterized by a tropical wet and dry savanna climate with consistently high temperatures throughout the year, an elevation ranging from approximately 150 to 300 m above sea level, and distinct monsoonal wet and dry seasons. The plant material was authenticated by P. Bunchalee, and a voucher specimen (N.TAESUK 001) was deposited at the Department of Biology, Faculty of Science, Mahasarakham University, Thailand.

### 3.3. Extraction

Fresh leaves of *G. vidalii* were cut into pieces and oven-dried at 55 °C. The dried leaves (280 g) were macerated in MeOH (10 L) at room temperature for 48 h and then filtered through a cheesecloth. The MeOH solution was concentrated under reduced pressure at 45 °C until the volume of the solution was ca. 1 L. After the concentrated solution was left overnight at room temperature, the precipitate formed at the bottom of the flask. The solution was then filtered through a Whatman No. 1 filter paper to separate the precipitate from the methanol solution and sequentially transferred to a 50 mL round-bottom flask and successively washed with hexane (25 mL × 2), EtOAc (25 mL × 2), and MeOH (25 mL × 2) by stirring for 30 min with each solvent. The combined washings from each solvent were added back to the original MeOH filtrate, while the remaining pale-yellow powder was collected as compound **1** (4.68 g).

The MeOH solution was concentrated under reduced pressure to afford the MeOH extract. A small portion of the extract was reserved for biological activity assays, while the remainder was suspended in water (500 mL) and successively partitioned with hexane (1 L × 2) and the hexane layer was concentrated to give hexane fraction (10.30 g). The aqueous layer was next partitioned with EtOAc (1 L ×3) and combined EtOAc layers were concentrated under reduced pressure until the volume of the solution was ca. 500 mL and left overnight at room temperature, resulting in the formation of a precipitate. The precipitate was collected by filtration through Whatman No. 1 filter paper and separated from the EtOAc solution. The precipitate was then washed with EtOAc (10 mL × 2), and MeOH (10 mL × 2) respectively by stirring for 30 min in a small container to obtain compound **1** (323.7 mg). The EtOAc solution was concentrated under reduced pressure to yield the EtOAc fraction (4.48 g).

**Scheme 1:**
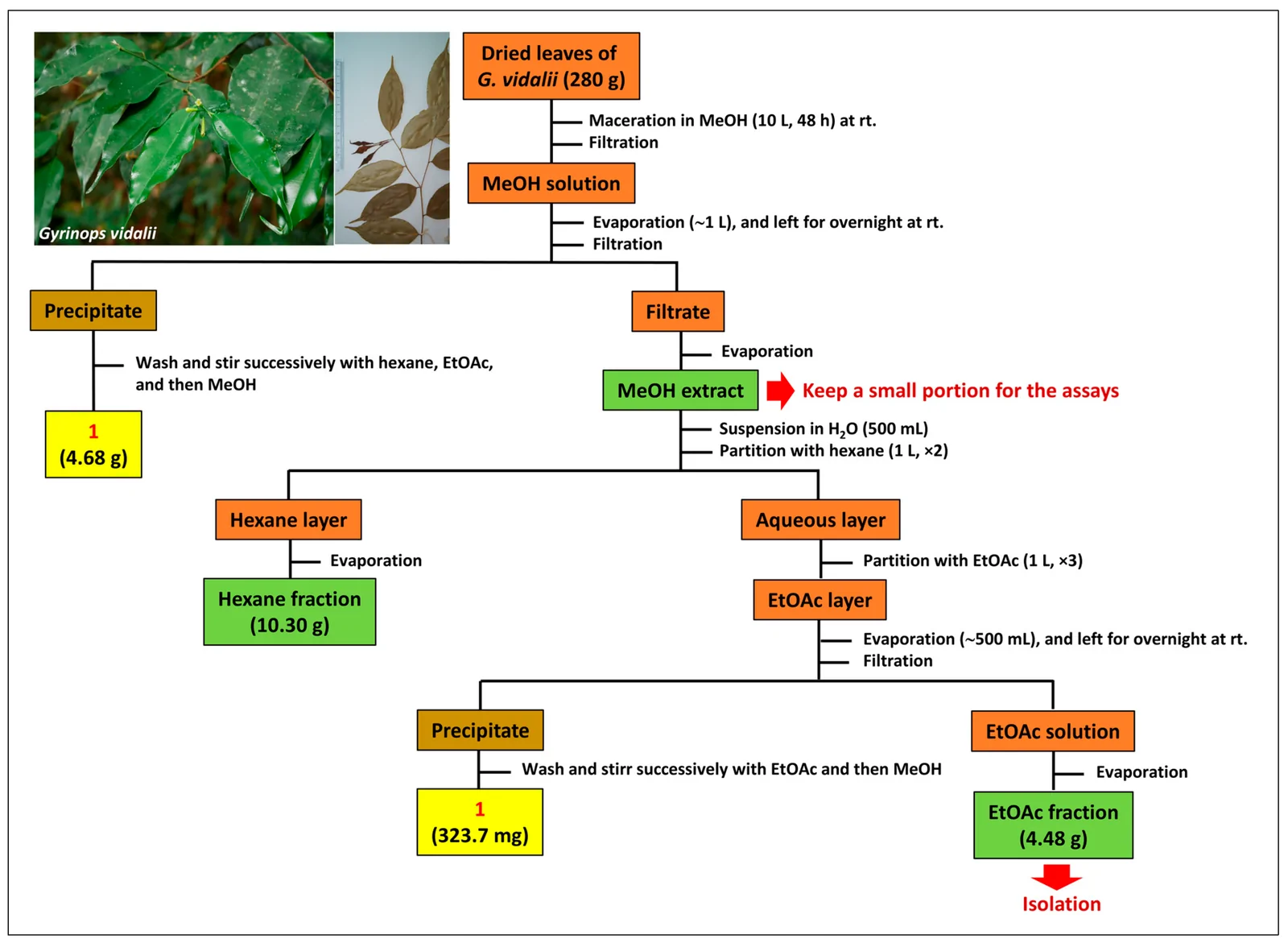
Extraction steps of *G. vidalii* leaves.

Based on the ^1^H NMR spectrum, which indicated a predominant aromatic compound bearing a sugar moiety, together with its antioxidant activity (DPPH assay, total phenolic and flavonoid contents), the EtOAc fraction was selected for further chemical investigation (Scheme 1).

### 3.4. Isolation

The EtOAc fraction was subjected to silica gel column chromatography (CC) (hexane–EtOAc, step gradient elution 80:20 to 0:100, and then EtOAc–MeOH, step gradient elution 90:10 to 30:70), yielding six main fractions (Fr.1–Fr.6). Fr.2 (168.3 mg) was fractionated by silica gel CC (hexane–EtOAc, step gradient elution 80:20 to 0:100, and then EtOAc–MeOH, step gradient elution 90:10 to 50:50) to afford two subfractions Fr.2A (72.7 mg) and Fr.2B (57.8 mg). Fr.2A was further purified by silica gel CC (hexane–EtOAc, step gradient elution 80:20 to 0:100, and then EtOAc–MeOH, step gradient elution 90:10 to 50:50) to yield **8** (light-yellow amorphous solid; 6.4 mg), **5** (light-yellow amorphous solid; 3.7 mg) and **9** (light-brown powder; 9.8 mg). Fr.2B was subjected to silica gel CC (hexane–EtOAc, 80:20 to 0:100, and EtOAc–MeOH, 90:10 to 30:70) to afford two sub-subfractions, Fr.2B1 (18.2 mg) and Fr.2B2 (22.7 mg). Fr.2B1 was further purified by Sephadex LH-20 CC (100% MeOH) to yield **7** (light-yellow oil; 3.1 mg) while **6** (light-yellow powder, 4.0 mg) was obtained from purification of Fr.2B2 by Sephadex LH-20 CC (100% MeOH). Fr.3 (216.5 mg; light-yellow crystals) was washed with a small volume of EtOAc twice in a small vial, and the resulting crystals were recrystallized from a mixed solvent of acetone/MeOH to afford **3** as light-yellow crystals (146.7 mg).

Fr.4 (2.61 g) was subjected to silica gel column CC (hexane–EtOAc, 60:40 to 0:100, and EtOAc–MeOH, 90:10 to 30:70) to obtain three subfractions Fr.4A (125.7 mg), Fr.4B (1.32 g), and Fr.4C (78.6 mg). Purification of Fr.4A by silica gel CC (hexane–EtOAc, 80:20 to 0:100, and EtOAc–MeOH, 90:10 to 30:70) afforded **3** (31.6 mg) and **4** (light-yellow powder; 26.6 mg). Purification of Fr.4B by Sephadex LH-20 CC (100% MeOH) to furnish **4** (604.7 mg), **9** (3.5 mg), and a sub-subfraction Fr.4B1 (112.6 mg). Fr.4B1 was fractionated by silica gel CC (hexane–EtOAc, 80:20 to 0:100, and EtOAc–MeOH, 90:10 to 30:70) yield **2** (light-yellow powder; 34.5 mg).

Fr.5 (1. 22 g) was fractionated by silica gel column CC (hexane–EtOAc, 60:40 to 0:100, and EtOAc–MeOH, 90:10 to 30:70) yielding four subfractions: Fr.5A (122.6 mg), Fr.5B (75.7 mg), Fr.5C (151.7 mg), and Fr.5D (230.8 mg). Fr.5B was further purified by silica gel CC (hexane–EtOAc, 60:40 to 0:100, and EtOAc–MeOH, 90:10 to 30:70) to afford **8** (3.2 mg). Purification of Fr.5C by silica gel CC (hexane–EtOAc, 60:40 to 0:100, and EtOAc–MeOH, 90:10 to 30:70) yielded **9** (20.8 mg) and **4** (24.6 mg).

**Scheme 2:**
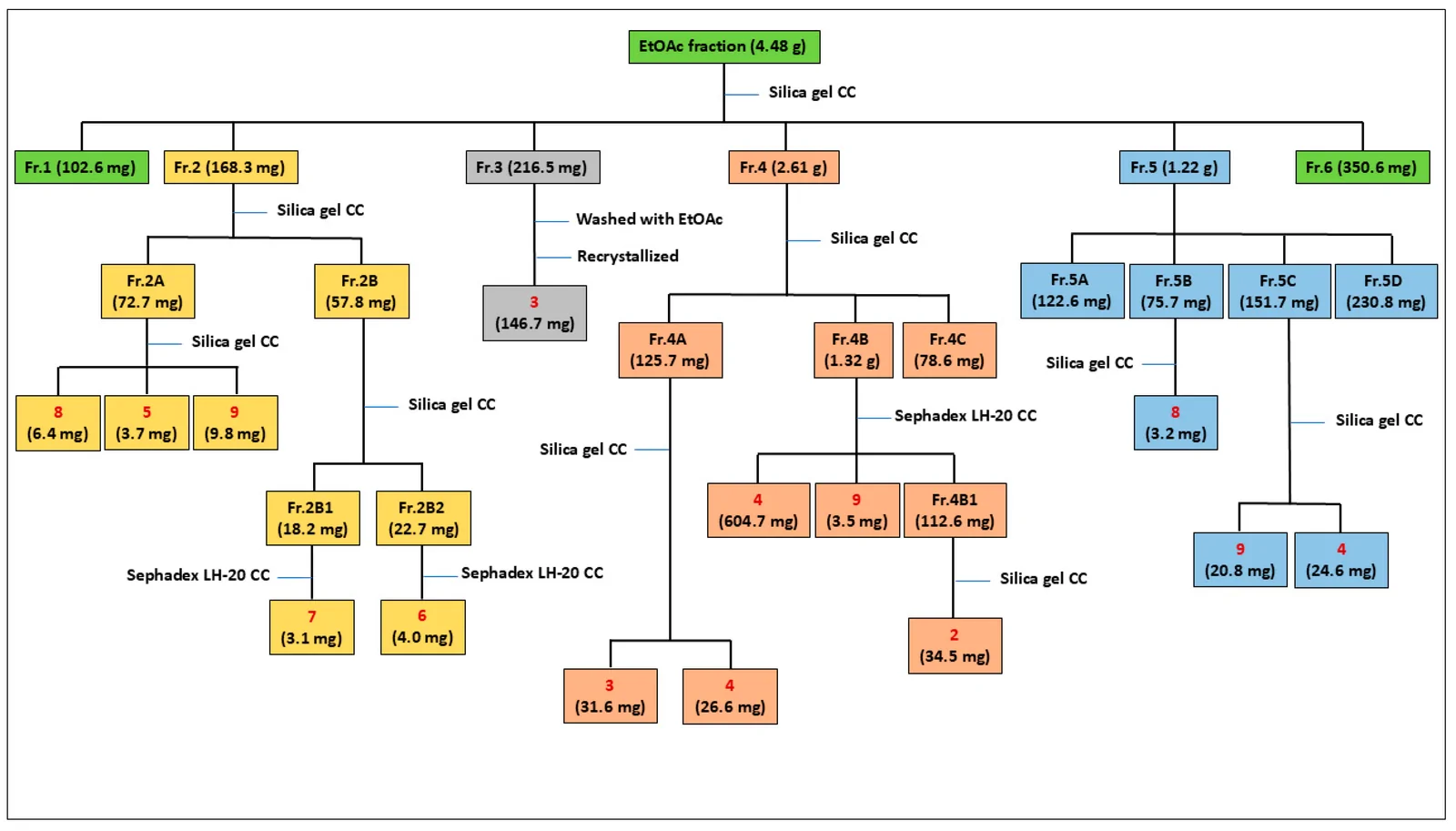
Isolation of the EtOAc fraction.

Based on the ^1^H NMR spectra, Fr.1 contained fatty substances, while Fr.6 displayed broad peak with no significant ^1^H NMR signals. These fractions were not further purified. The isolation process is summarized in Scheme 2.

### 3.5. Acid Hydrolysis of Compound ***3***

Compound **3** (7.6 mg) was dissolved in 10% H_2_SO_4_ (1.5 mL) and heated at 95 °C in an oil bath for 1 h. After cooling to room temperature, the reaction mixture was neutralized with NaHCO_3_ and extracted with EtOAc (1.0 mL, ×3). The combined organic layers were concentrated under reduced pressure to afford **3a** (5.7 mg, 75%).

### 3.6. Biological Activity Assay

#### 3.6.1. DPPH Radical Scavenging Activity

DPPH radical scavenging activity assay was performed as previously described by Promden et al. [78]. For the active samples showing >50% inhibition at 50 µg/mL, the SC_50_ was determined using a calibration curve within the linear range, plotting concentrations against % radical scavenging activity (%RSA). The experiments were performed in technical triplicate and reported as average ± standard deviation (SD). Ascorbic acid was used as a standard compound.

#### 3.6.2. Total Phenolic Content

The total phenolic content (TPC) of the plant extracts was determined using the Folin–Ciocalteu colorimetric method with slight modifications [79]. Briefly, 10 µL of appropriately diluted extract was mixed with 90 µL of 10% (*v*/*v*) Folin–Ciocalteu reagent in a 96-well microplate. After 5 min, 100 µL of 7% (*w*/*v*) sodium carbonate solution was added, and the final volume was adjusted to 200 µL. The mixture was incubated in the dark at room temperature for 30 min, and absorbance was measured at 765 nm using a microplate reader (FLUOstar^®^ Omega, BMG LABTECH, Ortenberg, Germany). Gallic acid was used to generate a calibration curve, and TPC was expressed as milligrams of gallic acid equivalents per gram of dry extract (mg GAE/g extract). All assays were performed in technical triplicate and reported as mean ± SD.

#### 3.6.3. Total Flavonoid Content

Total flavonoid content (TFC) was determined using the aluminum chloride colorimetric assay with slight modifications [79]. Briefly, 10 µL of extract solution was mixed with 40 µL of 0.25 M sodium nitrite and incubated for 5 min at room temperature, followed by addition of 50 µL of 0.15 M aluminum chloride and further incubation for 5 min. Subsequently, 100 µL of 1 M sodium hydroxide was added, and absorbance was measured at 510 nm using a microplate reader. Quercetin was used as the standard, and TFC was expressed as mg quercetin equivalents per g dry extract (mg QE/g extract). All assays were performed in technical triplicate and expressed as mean ± SD.

#### 3.6.4. α-Glucosidase Inhibitory Activity

The *α*-glucosidase inhibitory activity was evaluated as previously described [80]. For compounds showing >45% inhibition at 50 µg/mL, IC_50_ values were determined from concentration–response curves (3.125–100 µg/mL). Acarbose served as the standard inhibitor. All assays were performed in technical triplicate and expressed as mean ± SD.

### 3.7. Computational Method

The binding modes of compounds **3a** and **5** toward *α*-glucosidase from *Saccharomyces cerevisiae* were predicted using molecular docking calculation. The enzyme structure used in this study was adopted from the homology model developed in our previous work [81]. Briefly, the amino acid sequence of *α*-glucosidase (UniProt ID: P53051, MALX3_YEAST) was obtained from UniProtKB and submitted to the SWISS-MODEL server [82] for structure prediction using oligo-1,6-glucosidase from *S. cerevisiae* as a template. The resulting model was evaluated using PROCHECK, MolProbity, VERIFY-3D, and ERRAT, and showed satisfactory stereochemical and structural quality suitable for docking calculations, as previously reported [81].

Since the experimentally determined three-dimensional structure of *Saccharomyces cerevisiae α*-glucosidase (UniProt P53051, MALX3_YEAST) is not available, a homology model developed in our previous study was employed. The model was constructed using the crystal structure of *S. cerevisiae* isomaltase (PDB ID: 3A4A) as the template because of its high structural similarity to the target enzyme and was subsequently validated prior to docking calculations.

The three-dimensional structures of **3a** and **5** were obtained from the PubChem database and subjected to geometry optimization at the HF/6-31G(d,p) level using Gaussian 09 [83]. Docking calculations were then performed using AutoDock Vina (version 1.2.0) [84]. The docking grid was centered at x = 21.69, y = −5.85, and z = 23.83 with dimensions of 20 × 20 × 20 Å^3^ covering the protein binding pocket included the residues Asp69, His112, Arg213, Asp215, Glu277, His351, Asp352, and Arg442, which are associated with substrate recognition and catalytic activity [73,85]. During the molecular docking calculations, the protein target including the catalytic residues was kept rigid while all rotatable bonds in the ligands were allowed to move freely during docking, following the standard AutoDock Vina protocol.

To validate the docking protocol, maltose, a well-characterized competitive inhibitor of *α*-glucosidase, was redocked into the modeled enzyme. The predicted binding pose was then compared with the crystallographic orientation of maltose in *S. cerevisiae* isomaltase (PDB ID: 3A4A ), confirming the reliability of the docking procedure [73] Protein–ligand binding conformations and intermolecular interactions were subsequently analyzed using BIOVIA Discovery Studio Visualizer 2021 (version 21.1) [74].

## 4. Conclusions

The present study represents the first phytochemical investigation of the leaves of *Gyrinops vidalii*, a critically endangered agarwood-producing species native to Thailand and Laos. Guided by antioxidant activity and ^1^H NMR spectrum , the EtOAc fraction was selected for phytochemical investigation, leading to the isolation of seven compounds together with two phenolic acids. Mangiferin (**1**) was identified as the major constituent, while iriflophenone 2-*O*-*α*-L-rhamnoside (**4**) was the second most abundant compound. The occurrence of mangiferin, benzophenone glycosides, and other metabolites previously reported from *Aquilaria* species provides additional evidence supporting the close evolutionary relationship between *Gyrinops* and *Aquilaria*.

Among the isolated compounds, mangiferin (**1**) exhibited potent DPPH radical scavenging activity, exceeding that of ascorbic acid and providing phytochemical support for the traditional medicinal use of *G. vidalii* in promoting overall health. In contrast, iriflophenone (**3a**) and 5,7,4′-trimethoxyflavone (**5**) showed promising *α*-glucosidase inhibitory activities. The potent *α*-glucosidase inhibitory activities of **3a** and **5**, together with the previously reported activity of **1**, are consistent with the traditional use of *G. vidalii* leaves for the treatment of diabetes. Molecular docking demonstrated favorable binding of both compounds within the catalytic pocket of *α*-glucosidase, while in silico drug likeness and ADMET analyses suggested acceptable oral drug-like properties and favorable predicted pharmacokinetic profiles. The *α*-glucosidase inhibitory activity and molecular docking results provide initial evidence to support their potential biological relevance.

As mentioned, *Gyrinops vidalii* is a species native to Thailand, and the plant material investigated was obtained from a single population. Therefore, further studies on chemical variation among different populations, seasons, and plant tissues are needed. *Gyrinops vidalii* is a critically endangered species; future research should also focus on conservation strategies, propagation methods, and sustainable utilization approaches to support the protection of this valuable plant resource.

## Figures and Tables

**Table 1:** Antioxidant activities of the extract and fractions from *G. vidalii* leaves.

Sample	DPPH (SC_50_; ug/mL)	Total Phenolic Content (GAE/g Extract)	Total Flavonoid Content (mg QE/g Extract)
MeOH extract	109.5 ± 9.06	76.6 ± 2.97	97.6 ± 4.69
Hexane fraction	>150	ND	ND
EtOAc fraction	47.2 ± 3.84	192.3 ± 8.39	313.3 ± 2.66

GAE = gallic acid equivalent; QE = quercetin equivalent; and ND = not determined.

**Table 2:** DPPH radical scavenging and *α*-glucosidase inhibitory activities of **1–5** and **3a**.

Compound	DPPH (SC_50_; μM)	*α*-Glucosidase Inhibition (IC_50_; μM)
**1**	19.35 ± 1.34	ND
**2**	ND	ND
**3**	ND	ND
**4**	ND	ND
**5**	ND	174.57 ± 3.76
**3a**	ND	100.06 ± 11.40
Ascorbic acid	41.90 ± 0.14	ND
Acarbose	-	237.13 ± 22.01

SC_50_ and IC_50_ values are expressed as the mean ± SD of three independent experiments. Only compounds showing >50% DPPH radical scavenging activity at 50 μg/mL or >45% *α*-glucosidase inhibition at 50 μg/mL in the initial screening assay (Table S12) are selected for SC_50_ or IC_50_ determination. ND = not determined.

## Data Availability

The original contributions presented in this study are included in the article. Further inquiries can be directed to the corresponding authors.

## Supplementary Materials

The following supporting information can be downloaded at https://www.mdpi.com/article/10.3390/plants15162474/s1, Figure S1: Docking validation of maltose; Figure S2: ^1^H NMR spectra of the EtOAc extract (in CD_3_OD): Figures S3–S5: NMR spectra of **1** (in DMSO-*d*_6_); Figures S6–S10: NMR spectra of **2** (in DMSO-*d*_6_); Figure S11: MS spectrum of **2**; Figures S12–S15: NMR spectra of **3** (in CD_3_OD); Figure S16: HRESIMS spectrum of **3**; Figures S17–S19: NMR spectra of **4** (in CD_3_OD); Figure S20: MS spectrum of **4**; Figures S21–S23: NMR spectra of **5** (in CDCl_3_); Figures S24–S25: NMR spectra of **6** (in CD_3_OD); Figures S26–S28: NMR spectra of **6** (in CDCl_3_); Figures S29–S32: NMR spectra of **7** (in CDCl_3_); Figure S33: MS spectrum of **7**; Figure S34: NMR spectrum of **8** (in CDCl_3_)**;** Figure S35: NMR spectrum of **9** (in CD_3_OD); Figures S36–S38: NMR spectra of **3a** (in DMSO-*d*_6_); Figures S39–S41: NMR spectra of **3a** (in acetone-*d*_6_). Table S1: NMR spectroscopic data of **1**; Table S2: NMR spectroscopic data of **2**; Table S3: NMR spectroscopic data of **3**; Table S4: NMR spectroscopic data of **4**; Table S5: NMR spectroscopic data of **5**; Table S6: NMR spectroscopic data of **6**; Table S7: NMR spectroscopic data of **7**; Table S8: NMR spectroscopic data of **8** and **9**; Table S9: NMR spectroscopic data of **3a**. Table S10: Lipinski’s Rule of 5 for **3a** and **5**. Table S11: Predicted ADMET properties of **3a** and **5**. Table S12: Preliminary biological screening results of compounds **1**–**5** and **3a**.
