# Supplementary material for: Phytochemical Investigation of Gyrinops vidalii Leaves and Evaluation of the α-Glucosidase Inhibitory Activity of the Isolated Metabolites and Molecular Docking of the Active Compounds
Source: Plants (Basel). 2026 Aug 15;15(16):2474. doi: 10.3390/plants15162474 (PMC13517060; doi:10.3390/plants15162474)
Supplement: Supplementary file 1 [file plants-15-02474-s001.zip › plants-4477246-supplementary.pdf]

Supplementary Information

Phytochemical Investigation of *Gyrinops vidalii* leaves, Evaluation of the *α*-Glucosidase Inhibitory Activity of the Isolated Metabolites and Molecular Docking of the Active Compounds

Siriwan Srisit[1], Thittaya Ketnad[1], Khemika Singmahan[1], Pasakorn Bunchalee[2], Worrawat Promden,[3] Panawan Moosophon[4], Vanida Choomuenwai[5], Bunleu Sungthong[6], Nadtanet Nanthaboot[1], Anake Kijjoa[7,*], Prapairat Seephonkai[1,*]

[1] Department of Chemistry and Center of Excellence for Innovation in Chemistry (PERCH-CIC), Faculty of Science, Mahasarakham University, Khamriang, Kantarawichai, Maha Sarakham 44150, Thailand; srisitsiriwan@gmail.com, tittayakatnad@gmail.com, khemika15728@gmail.com, nadtanet@gmail.com, prapairat.s@msu.ac.th

[2] Department of Biology, Faculty of Science, Mahasarakham University, Khamriang, Kantarawichai, Maha Sarakham 14450, Thailand; pasakorn.b@msu.ac.th

[3] Division of General Science, Faculty of Education, Buriram Rajabhat University, Buriram 31000, Thailand; worrawat.pd@bru.ac.th

[4] Department of Chemistry and Center of Excellence for Innovation in Chemistry, Khon Kaen University, Khon Kaen 40002, Thailand; mpanaw@kku.ac.th

[5] Department of Chemistry, Faculty of Science and Technology, Nakhon Ratchasima Rajabhat University, Nakhon Ratchasima 30000, Thailand; vanida.c@nrru.ac.th

[6] Faculty of Pharmacy, Mahasarakham University, Maha Sarakham 44150, Thailand; bunleu.s@msu.ac.th

[7] School of Medicine and Biomedical Sciences Abel Salazar (ICBAS) and CIIMAR, Universidade do Porto, Rua de Jorge Viterbo Ferreira, 228, 4050-313 Porto, Portugal; ankijjoa@icbas.up.pt

* Correspondence: ankijjoa@icbas.up.pt and prapairat.s@msu.ac.th

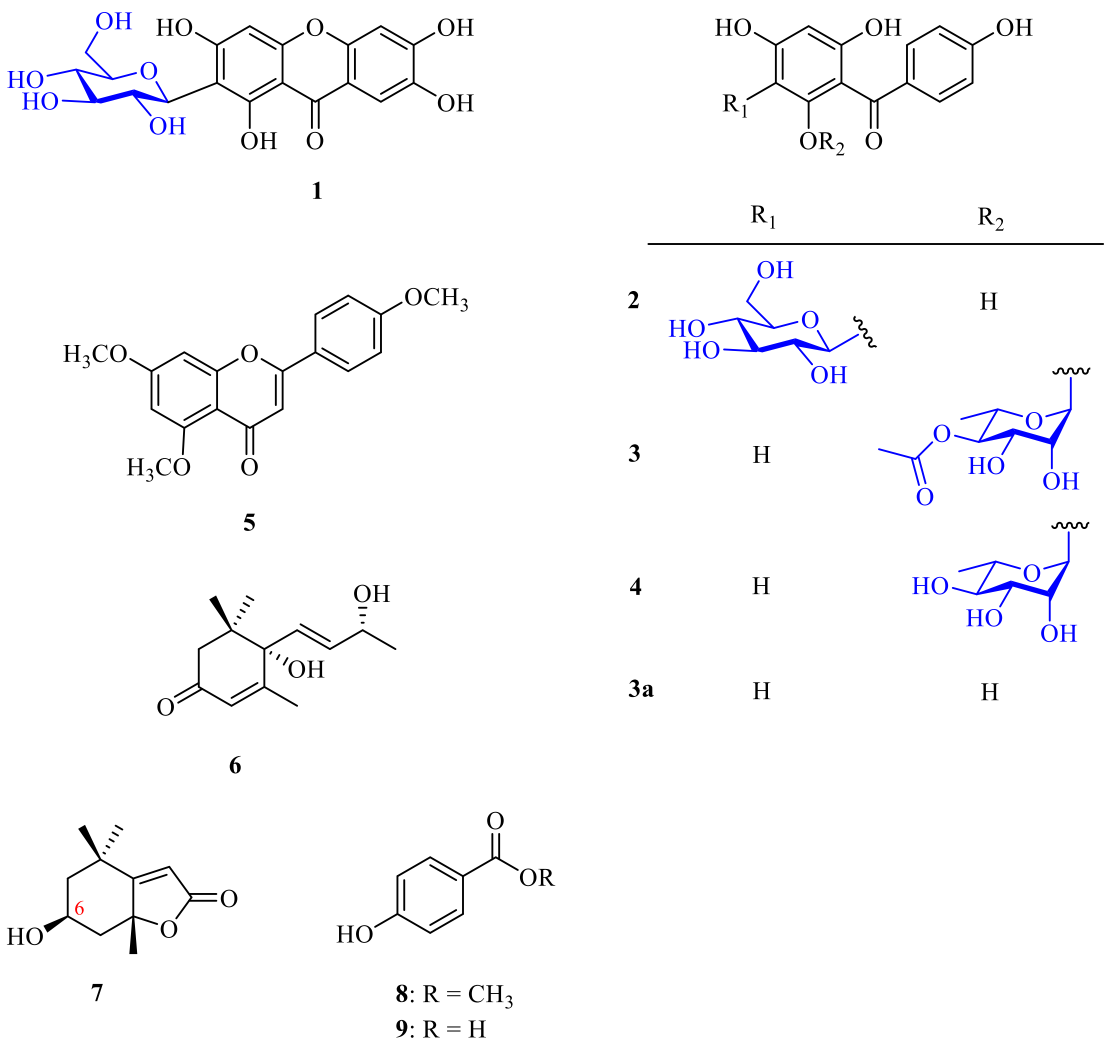

Structures of the compounds **1**–**9** and **3a**.

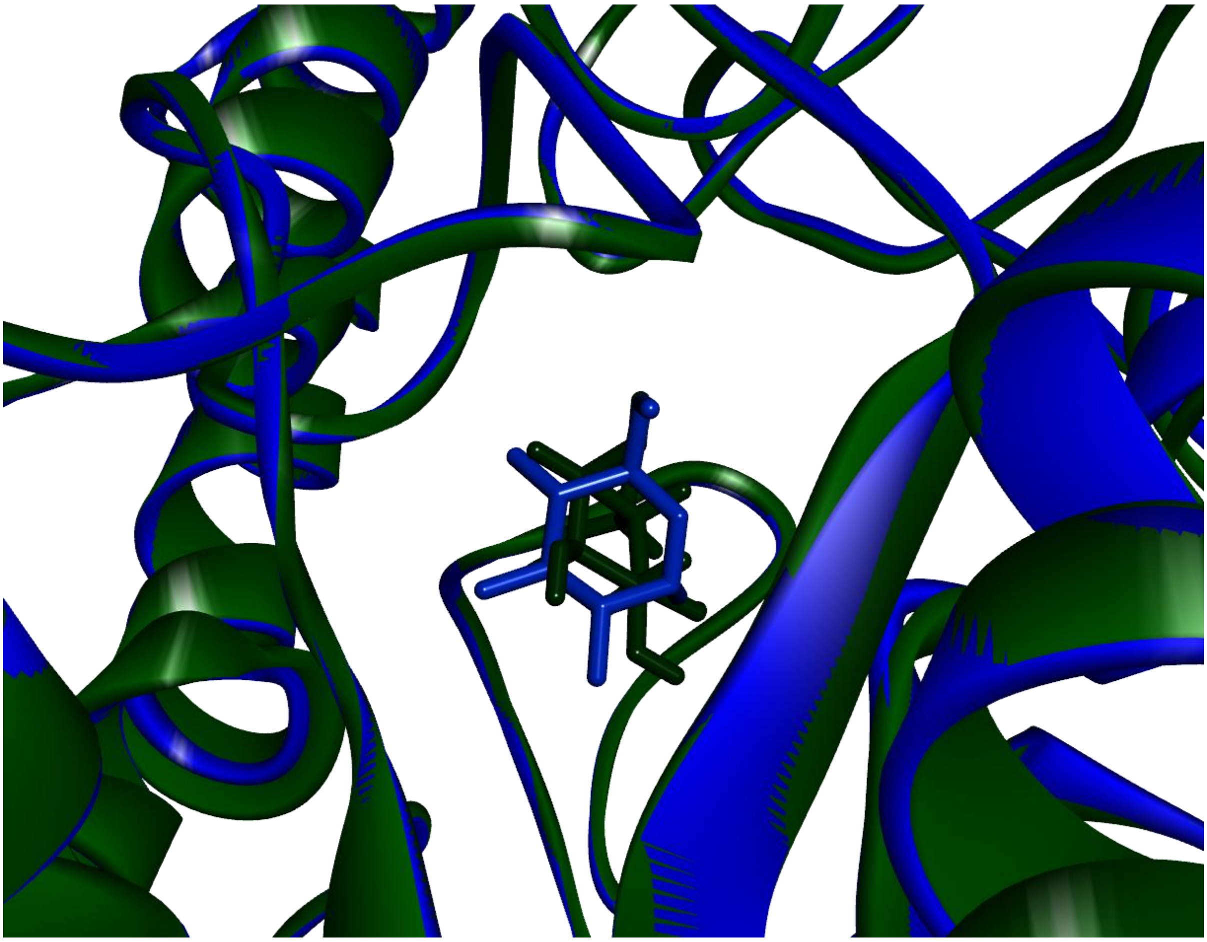

Figure S1. Superposition of protein structures between the modeled α-glucosidase (green) and isomaltase from *S. cerevisiae* (3A4A.pdb, blue). The redocked pose (green) of maltose in α-glucosidase and the crystallographic pose (blue) of maltose in isomaltase are in stick representation.

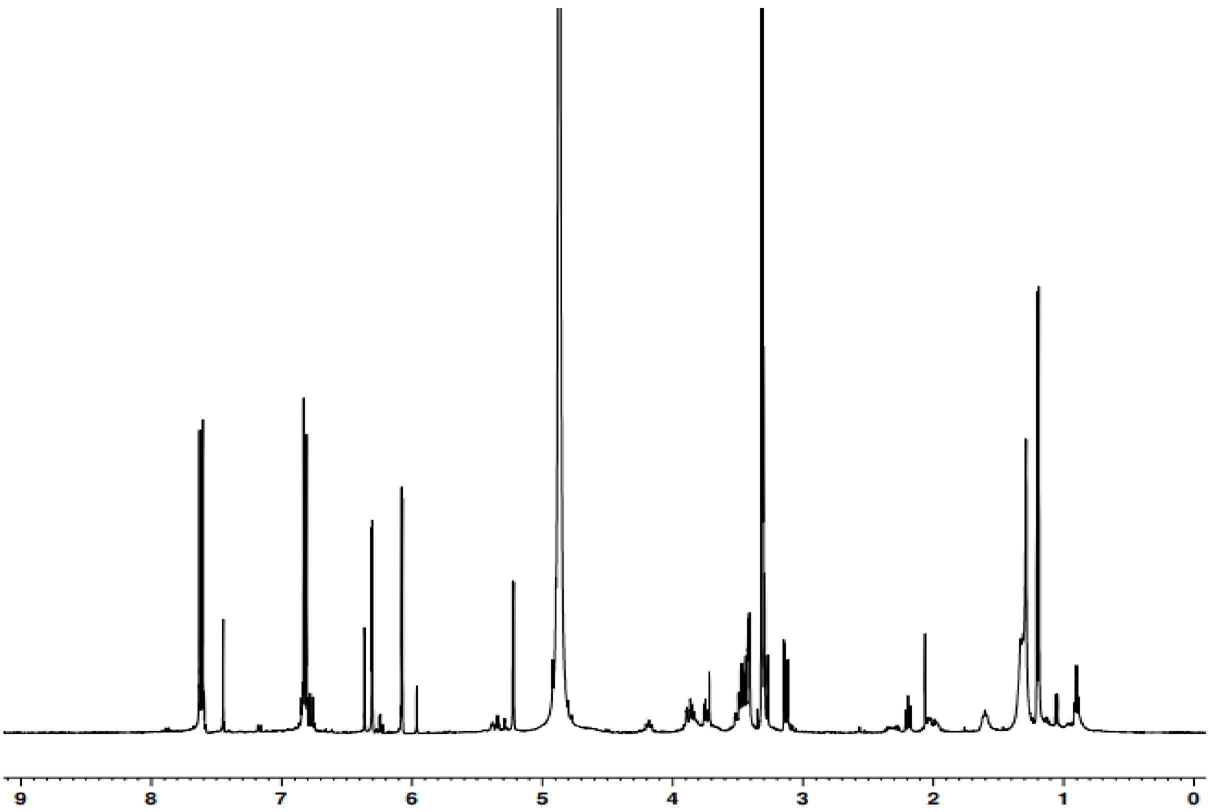

Figure S2. ^{1}H NMR spectrum (400 MHz) of the EtOAc extract in CD_3OD.

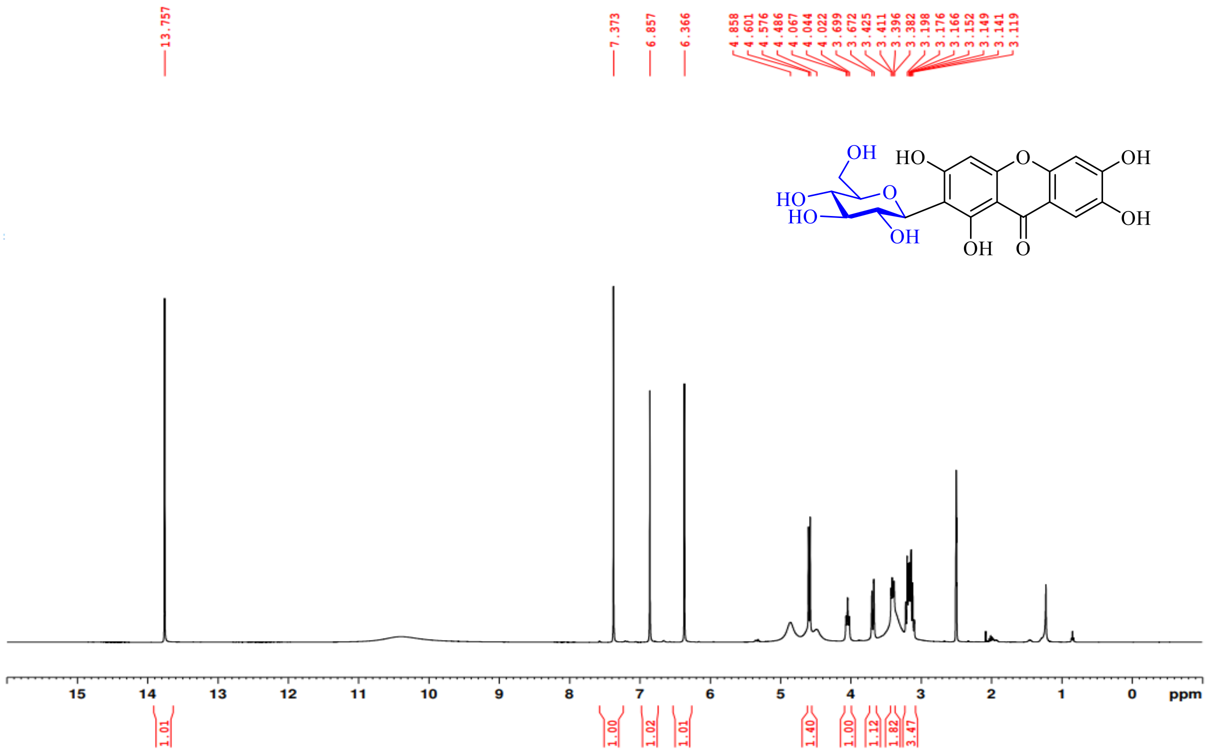

Figure S3. ^{1}H NMR spectrum (400 MHz) of **1** in DMSO-d_6.

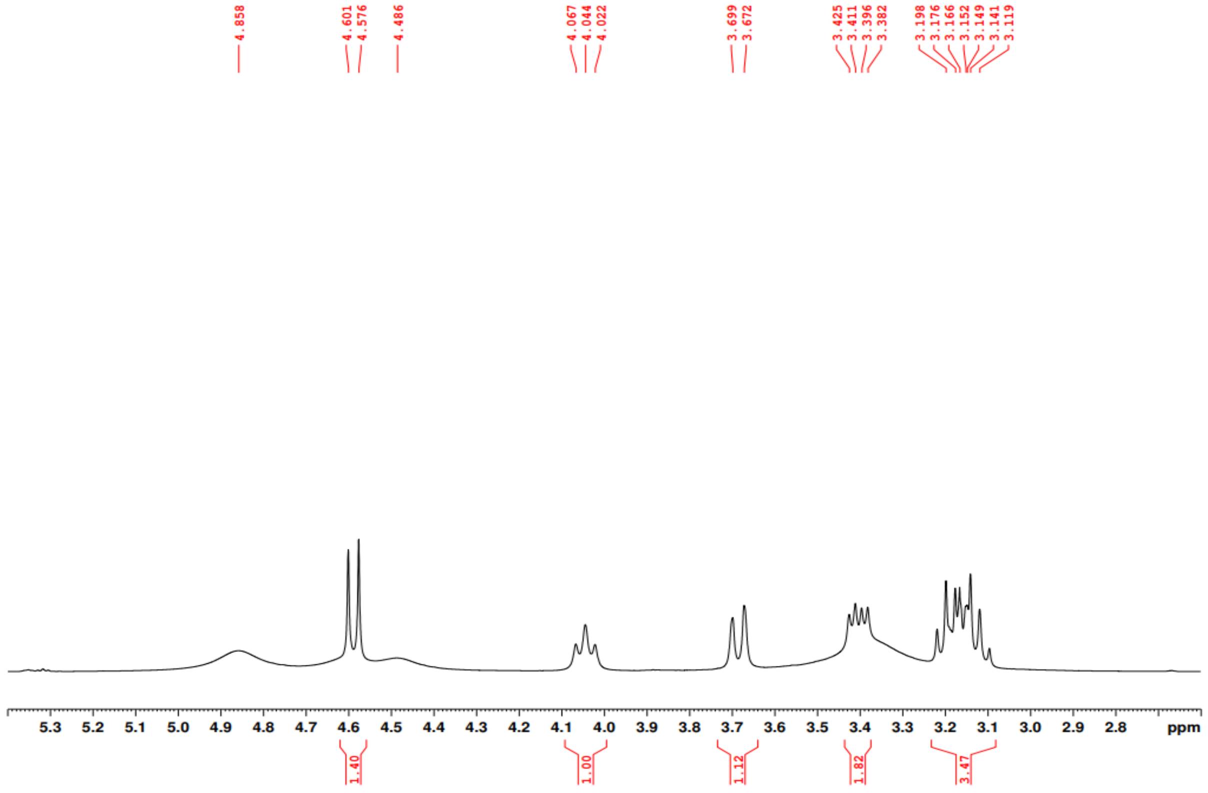

Figure S4. ^{1}H NMR spectrum (400 MHz) (expansion 1) of **1** in DMSO-d_6.

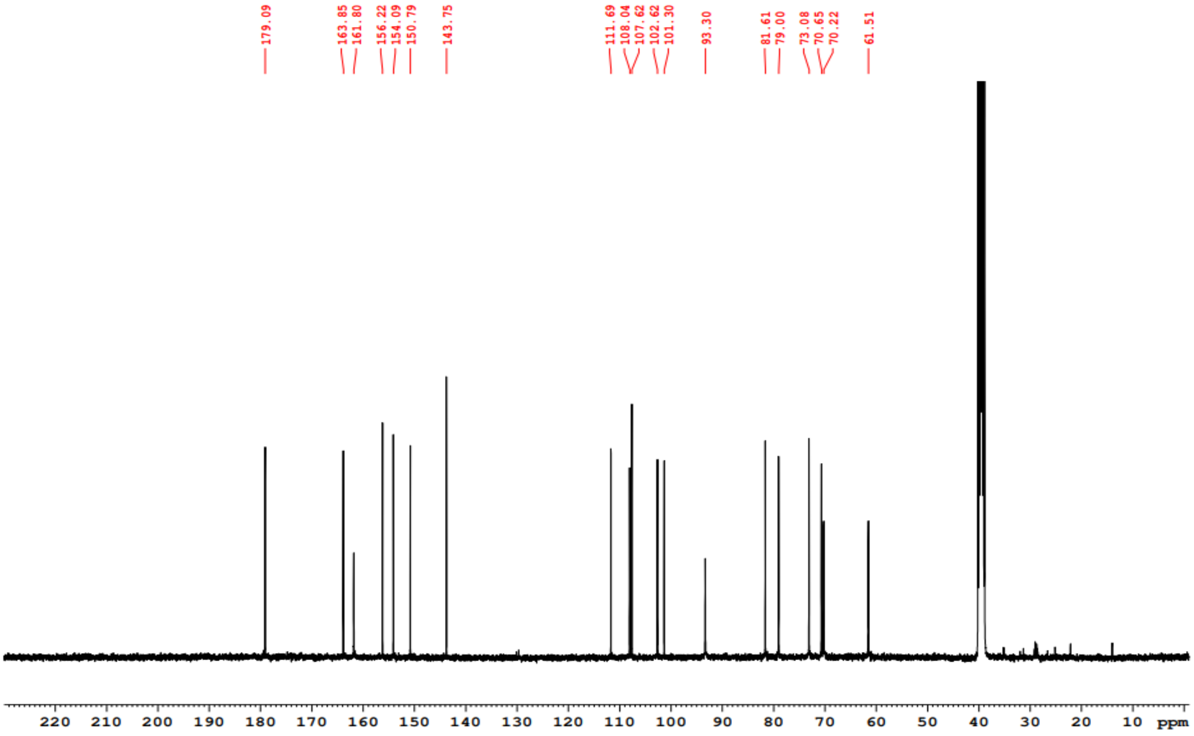

Figure S5. ^{13}C NMR spectrum (100 MHz) of **1** in DMSO-d_6.

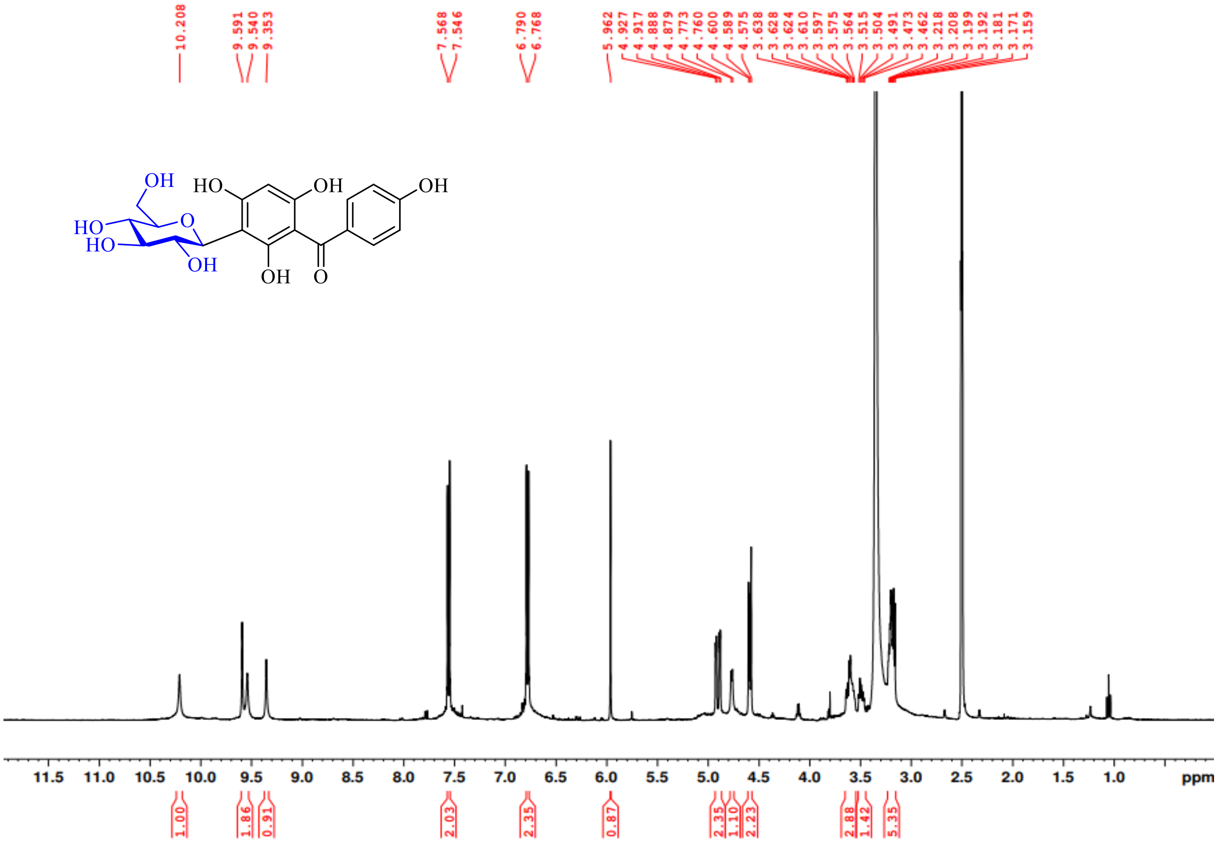

Figure S6. ^{1}H NMR spectrum (400 MHz) of **2** in DMSO-d_6.

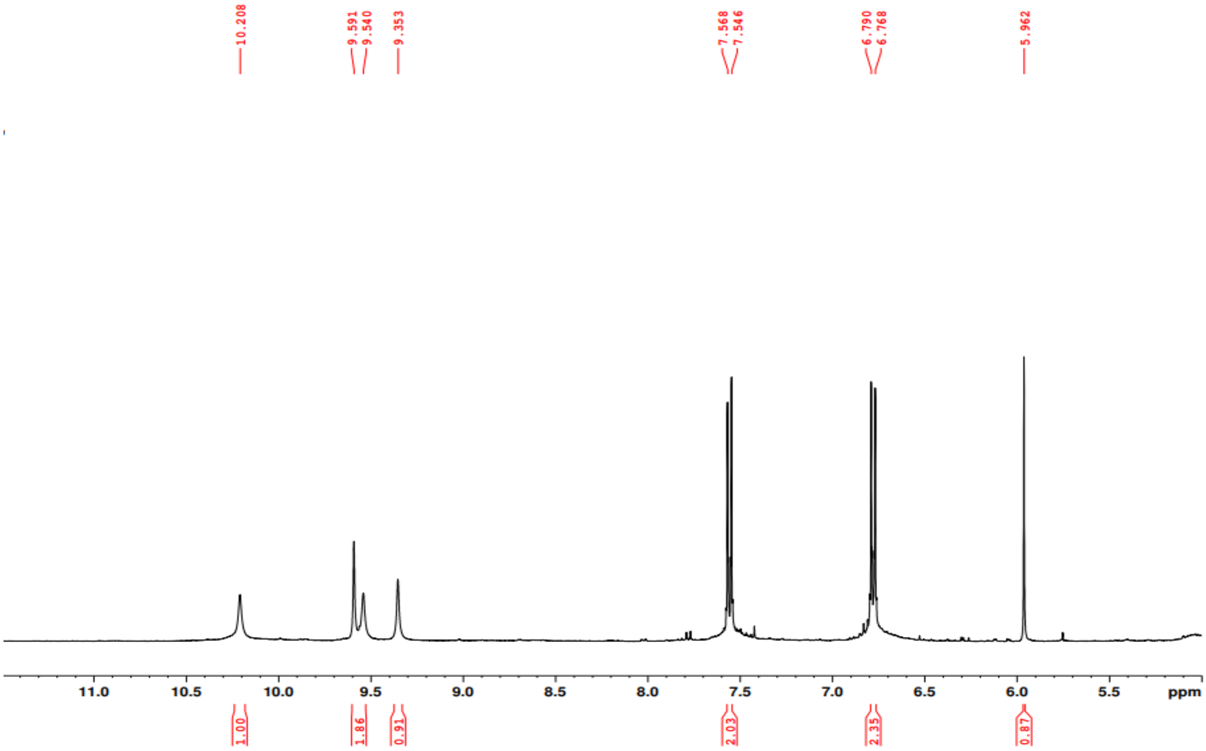

Figure S7. ^{1}H NMR spectrum (400 MHz) (expansion 1) of **2** in DMSO-*d*6.

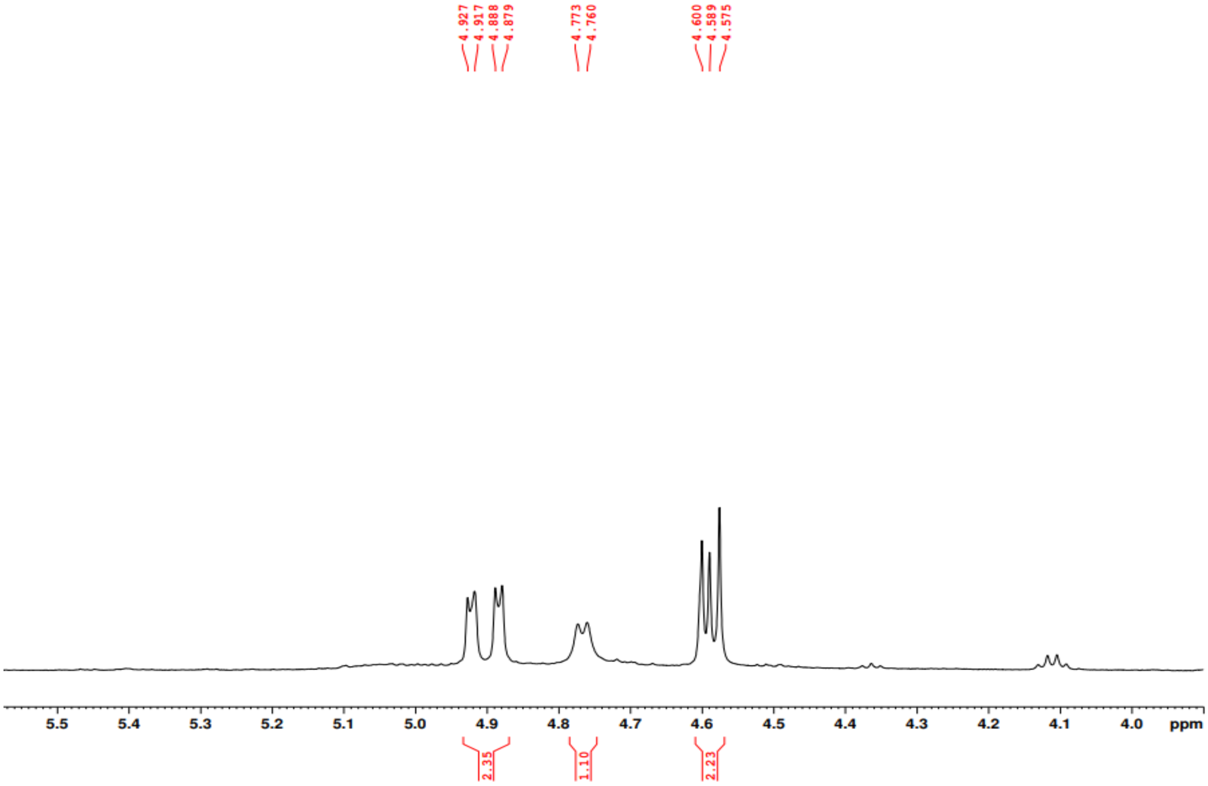

Figure S8. ^{1}H NMR spectrum (400 MHz) (expansion 2) of **2** in DMSO-*d*6.

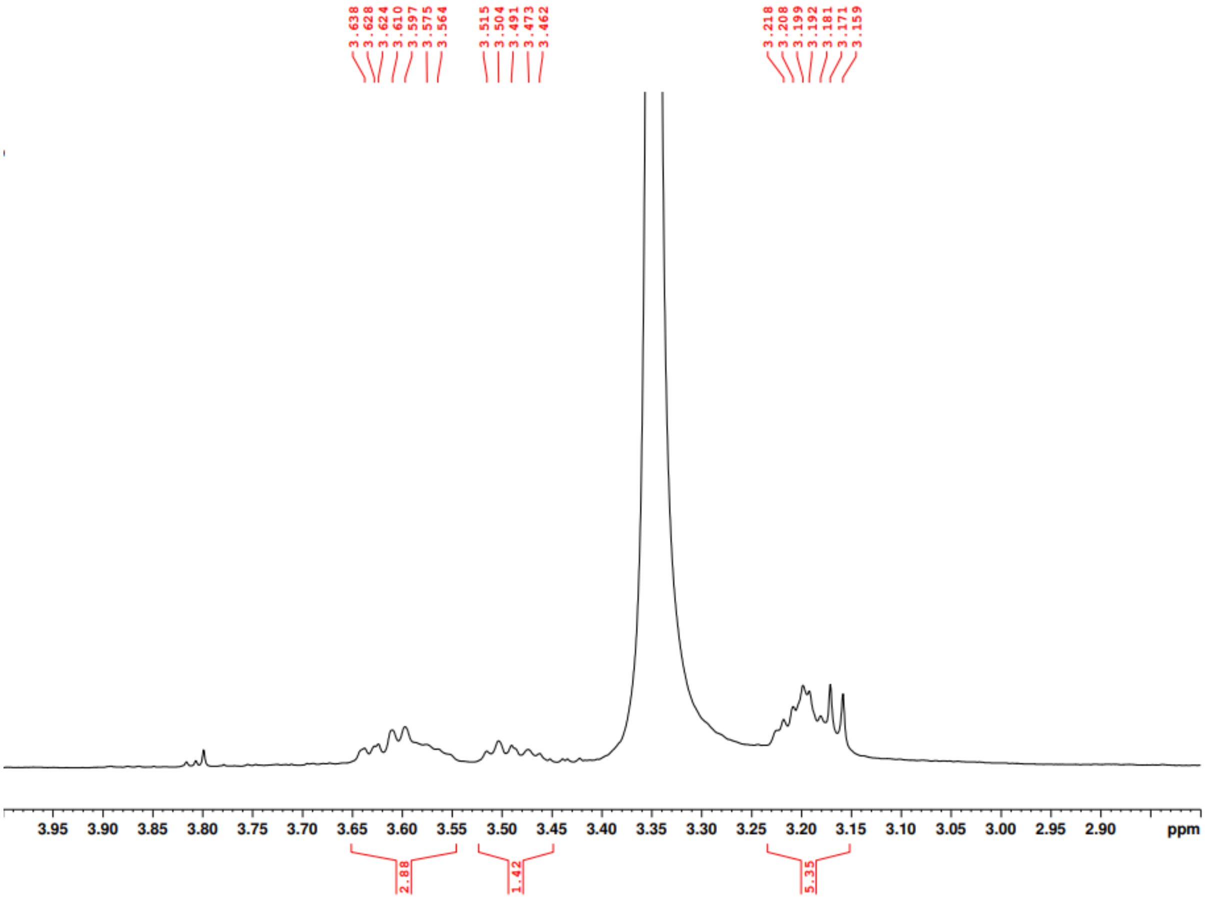

Figure S9. ^{1}H NMR spectrum (400 MHz) (expansion 3) of **2** in DMSO-d_6.

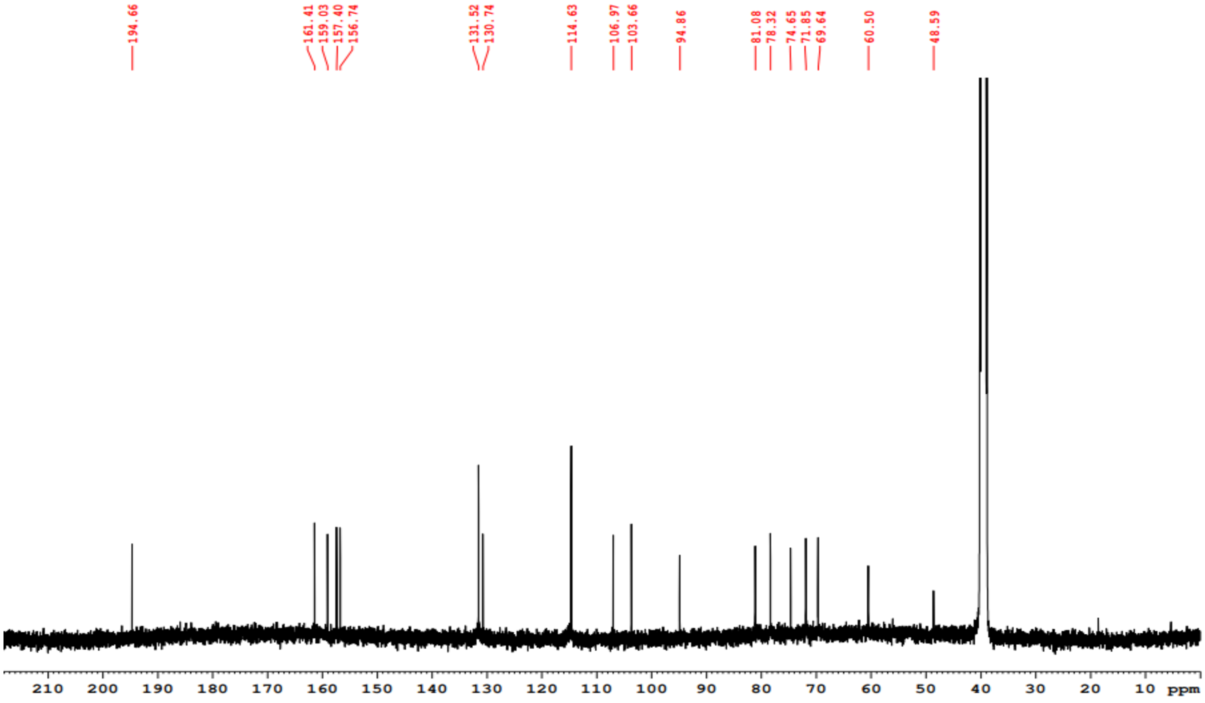

Figure S10. ^{13}C NMR spectrum 100 MHz) (expansion 2) of **2** in DMSO-d_6.

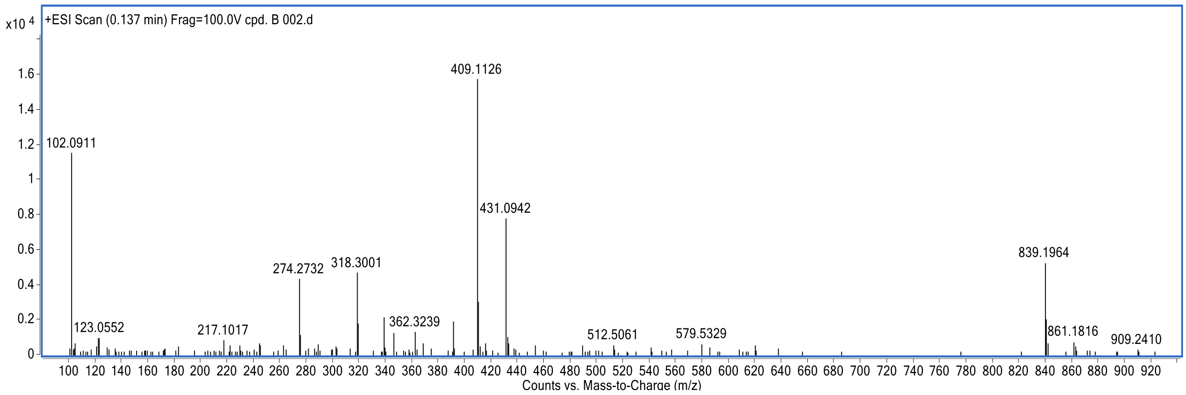

Figure S11. MS spectrum (positive mode) of **2**.

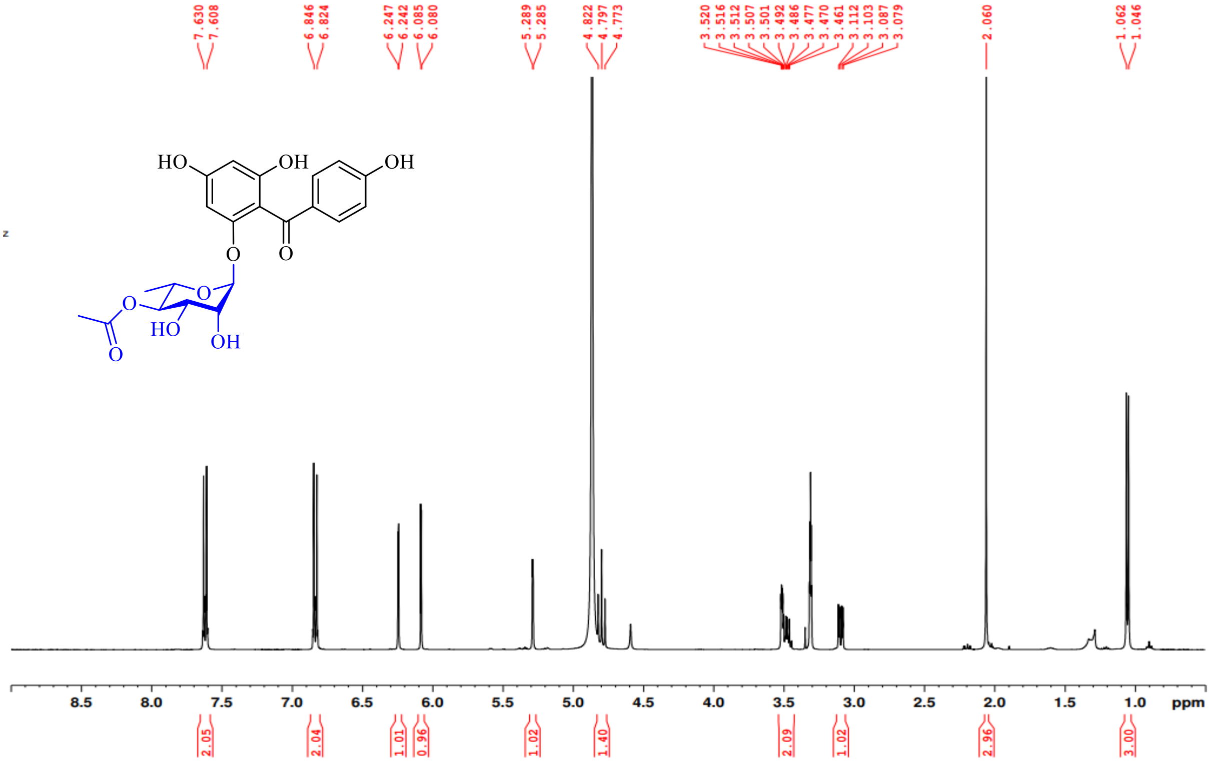

Figure S12. ^{1}H NMR spectrum (400 MHz) of **3** in CD_3OD.

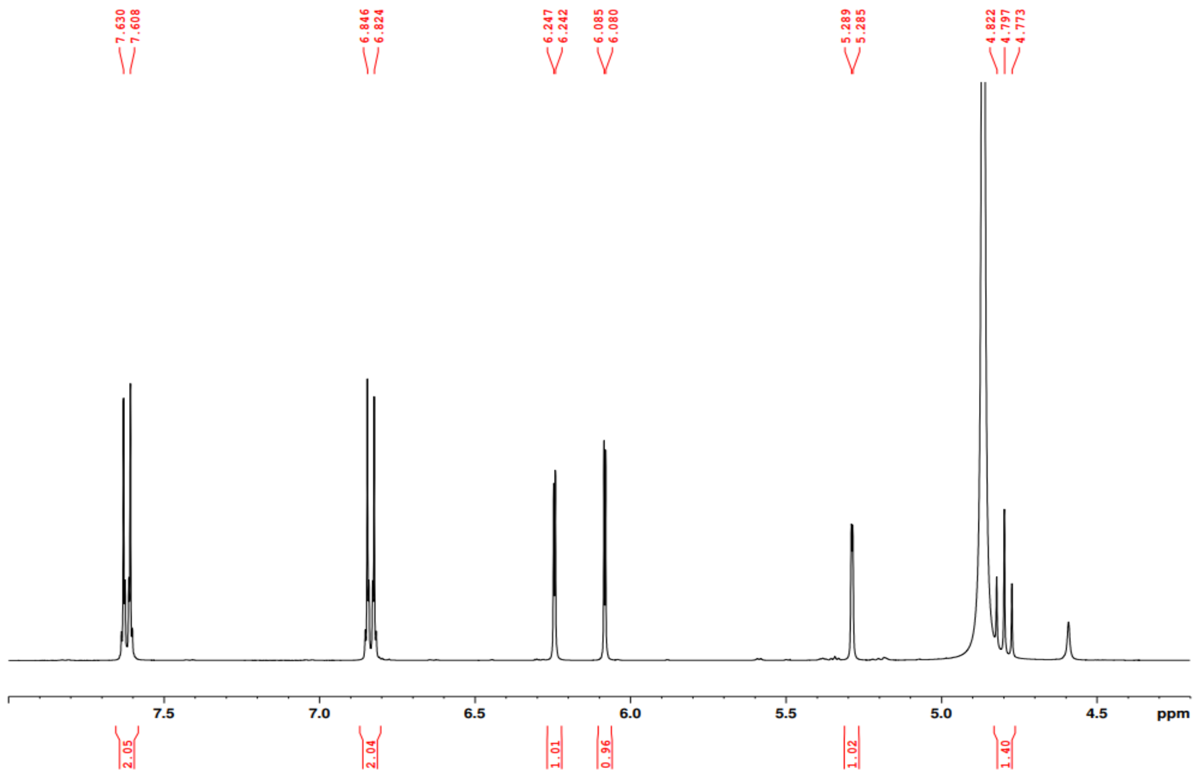

Figure S13. ^{1}H NMR spectrum (400 MHz) (expansion 1) of **3** in CD_3OD.

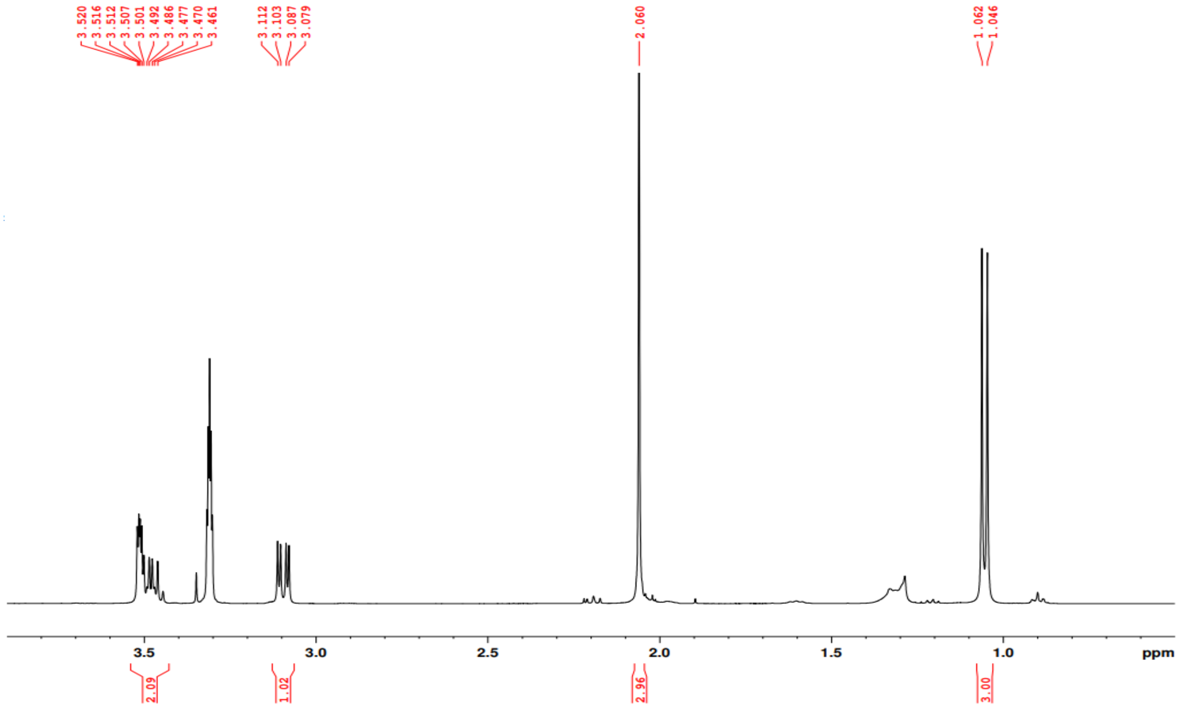

Figure S14. ^{1}H NMR spectrum (400 MHz) (expansion 2) of **3** in CD_3OD.

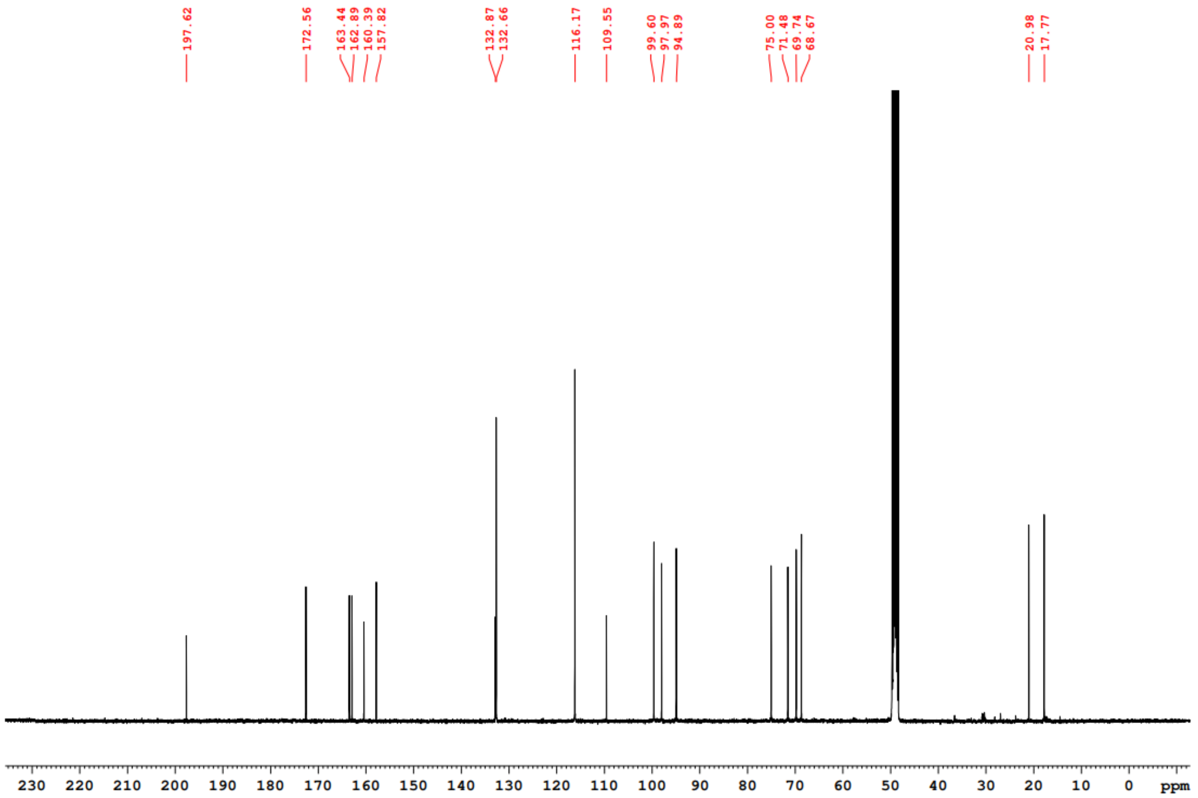

Figure S15. ^{13}C NMR (100 MHz) spectrum of **3** in CD_3OD.

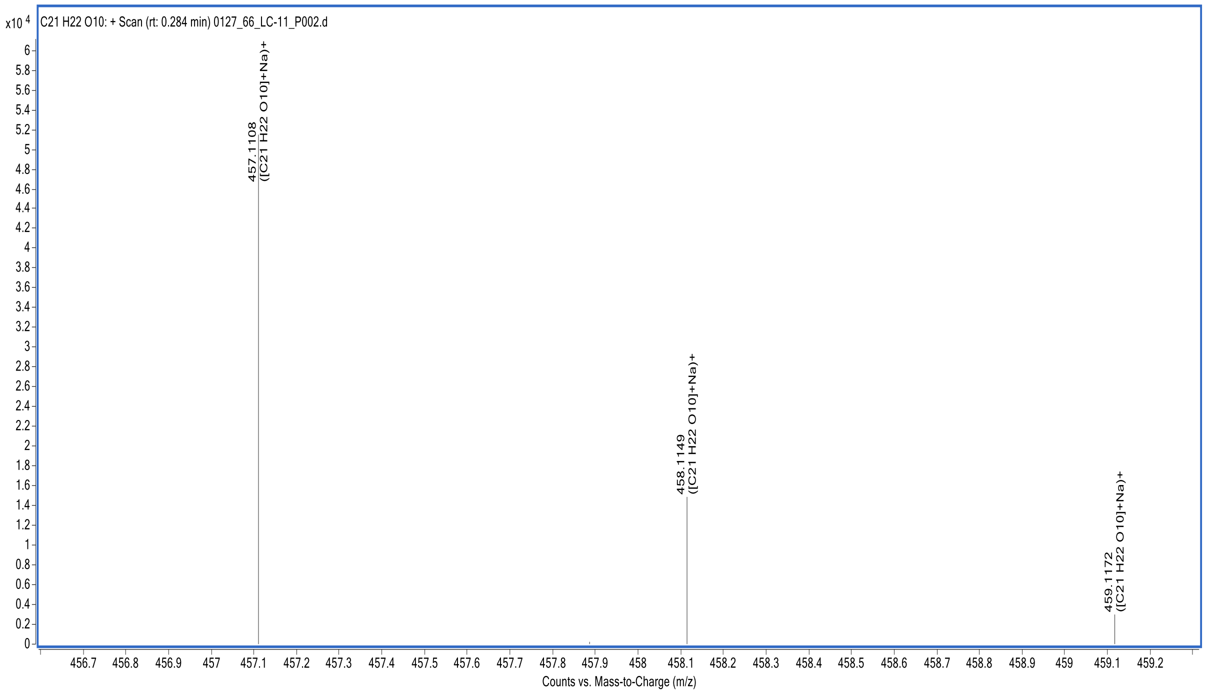

Figure S16. HRESIMS spectrum (positive mode) of **3**.

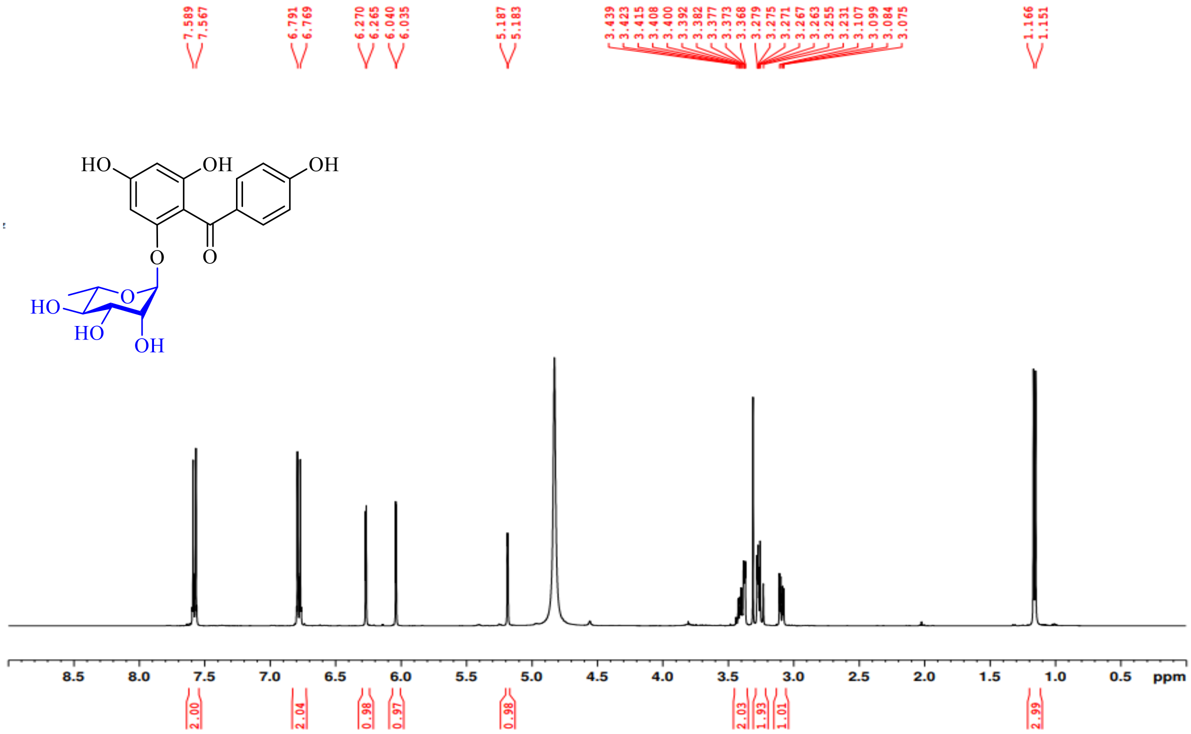

Figure S17. ^{1}H NMR spectrum (400 MHz) of **4** in CD_3OD.

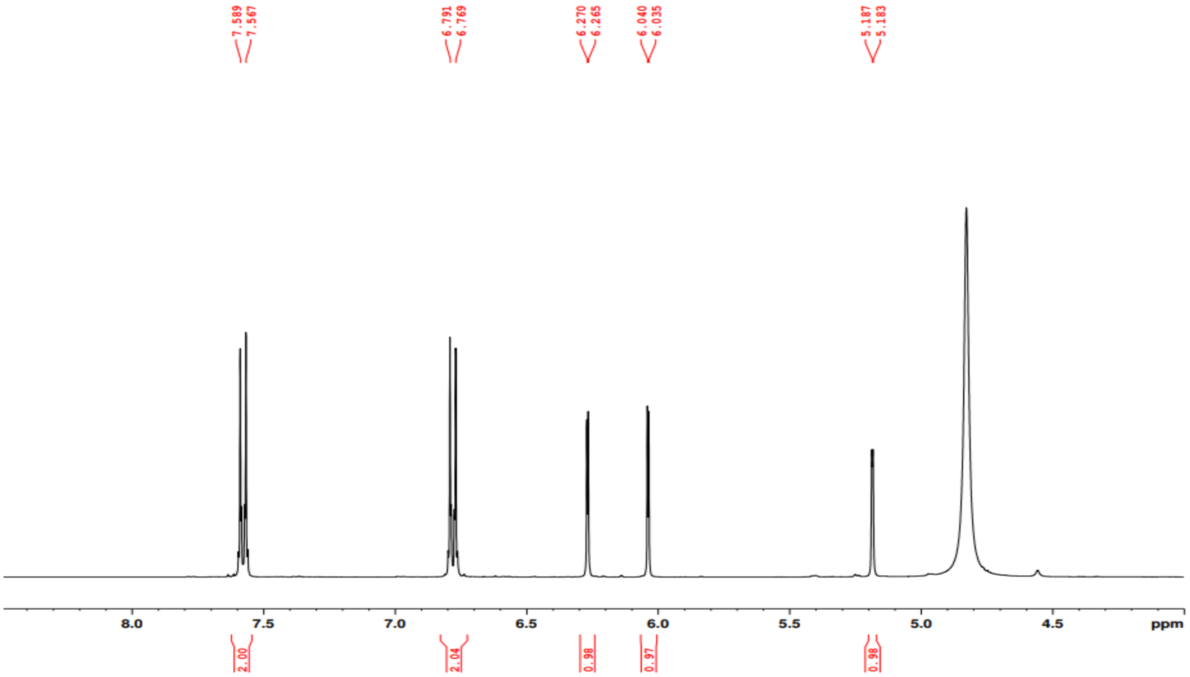

Figure S18. ^{1}H NMR spectrum (400 MHz) (expansion 1) of **4** in CD_3OD.

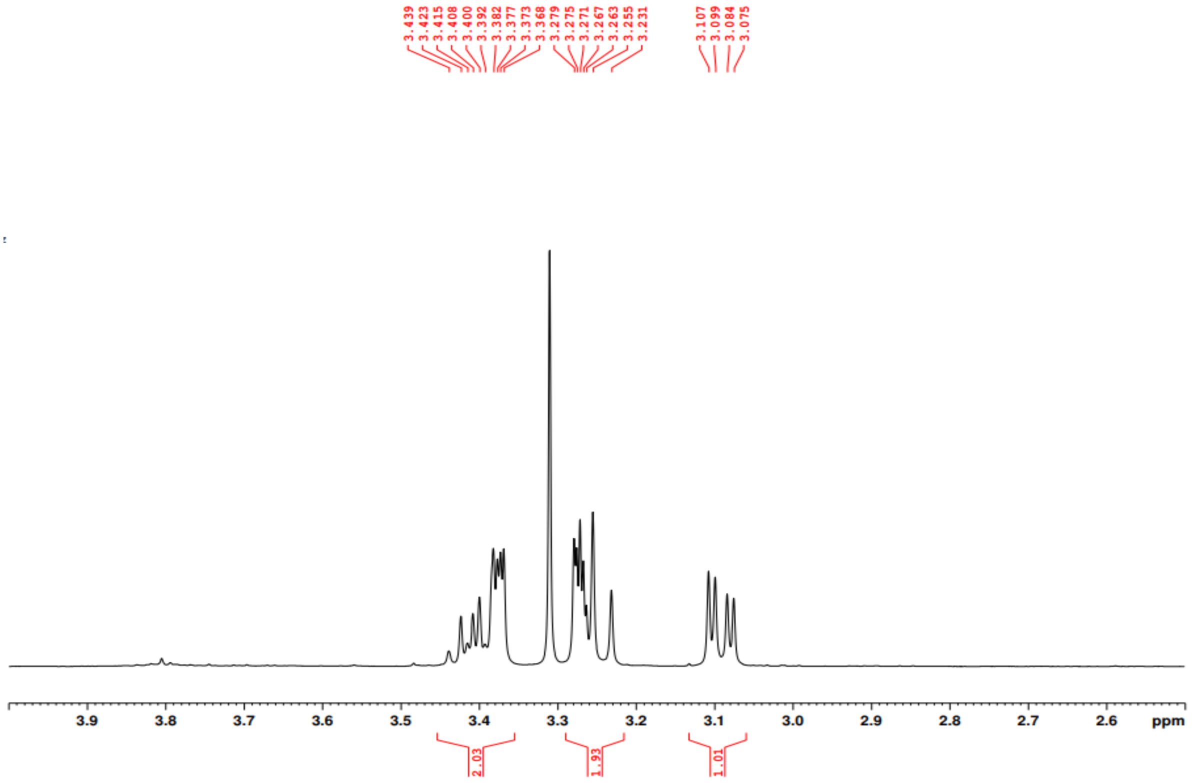

Figure S19. ^{1}H NMR spectrum (400 MHz) (expansion 2) of **4** in CD_3OD.

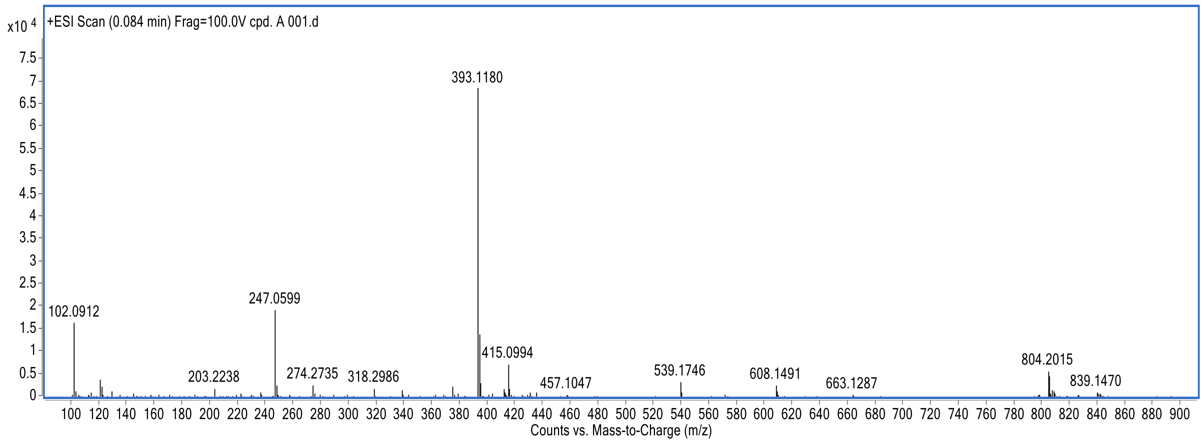

Figure S20. MS spectrum (positive mode) of **4**.

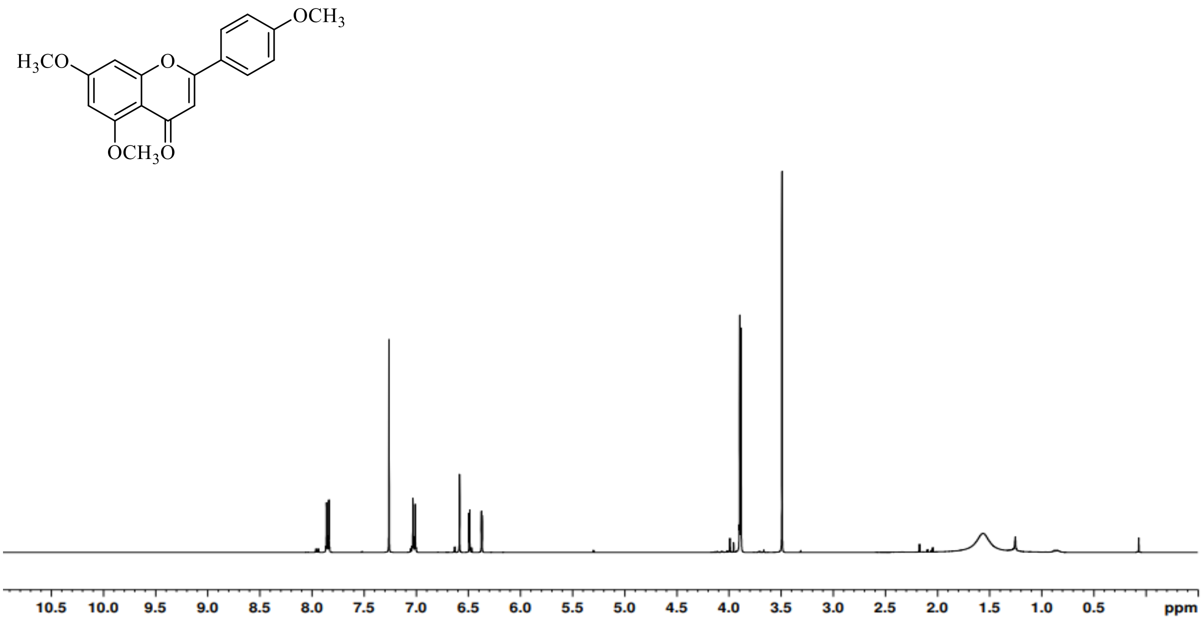

Figure S21. ^{1}H NMR spectrum (400 MHz) of **5** in $CDCl_3$.

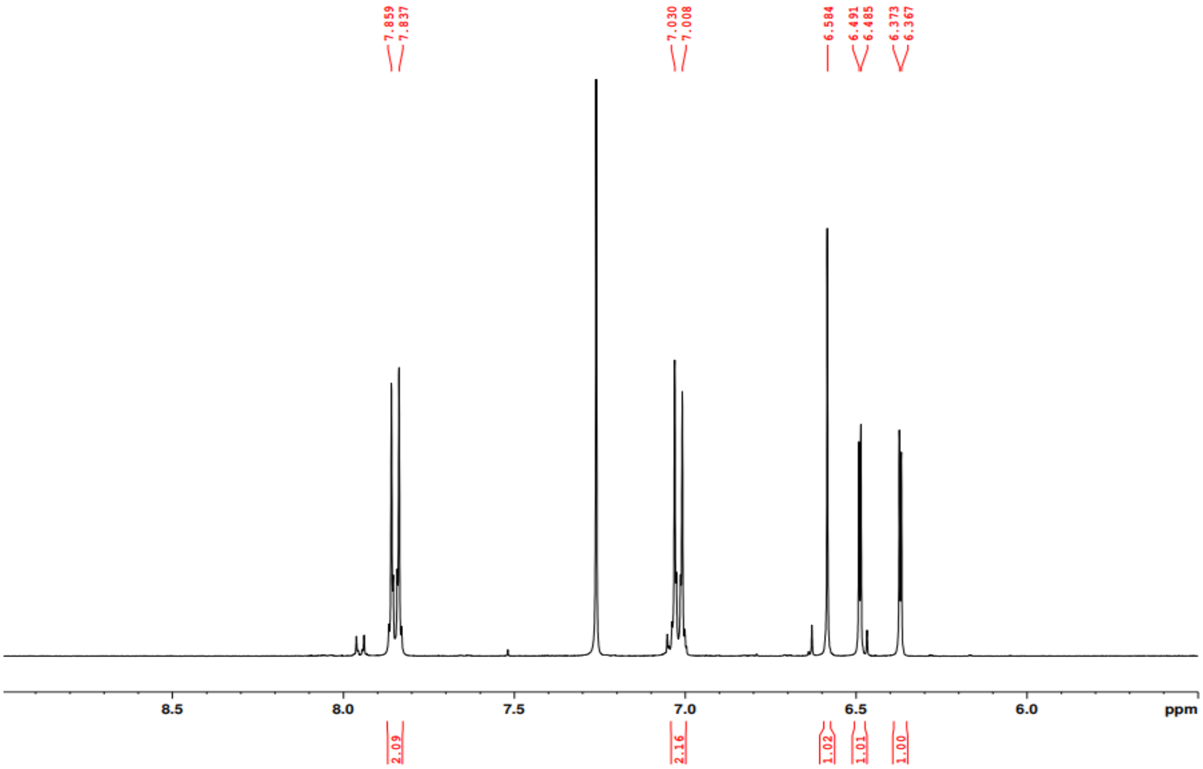

Figure S22. ^{1}H NMR spectrum (400 MHz) (expansion 1) of **5** in $CDCl_3$.

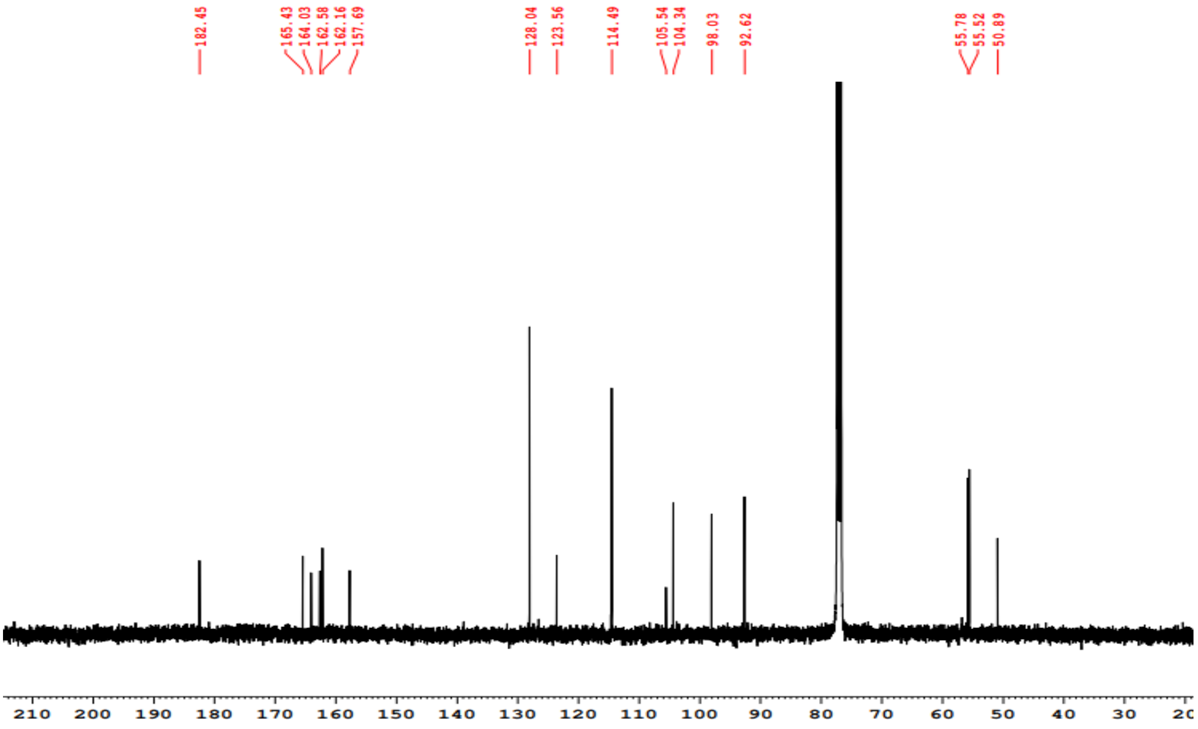

Figure S23. ^{13}C NMR spectrum (100 MHz) of **5** $CDCl_3$.

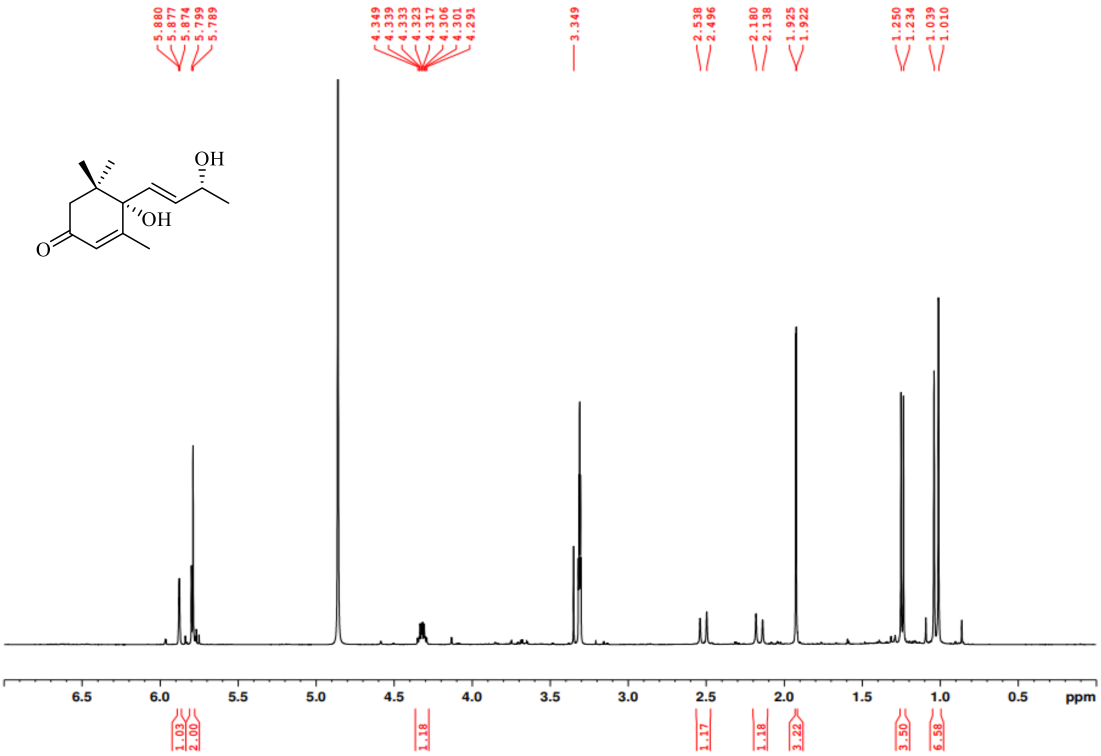

Figure S24. ^{1}H NMR spectrum (400 MHz) of **6** in CD_3OD.

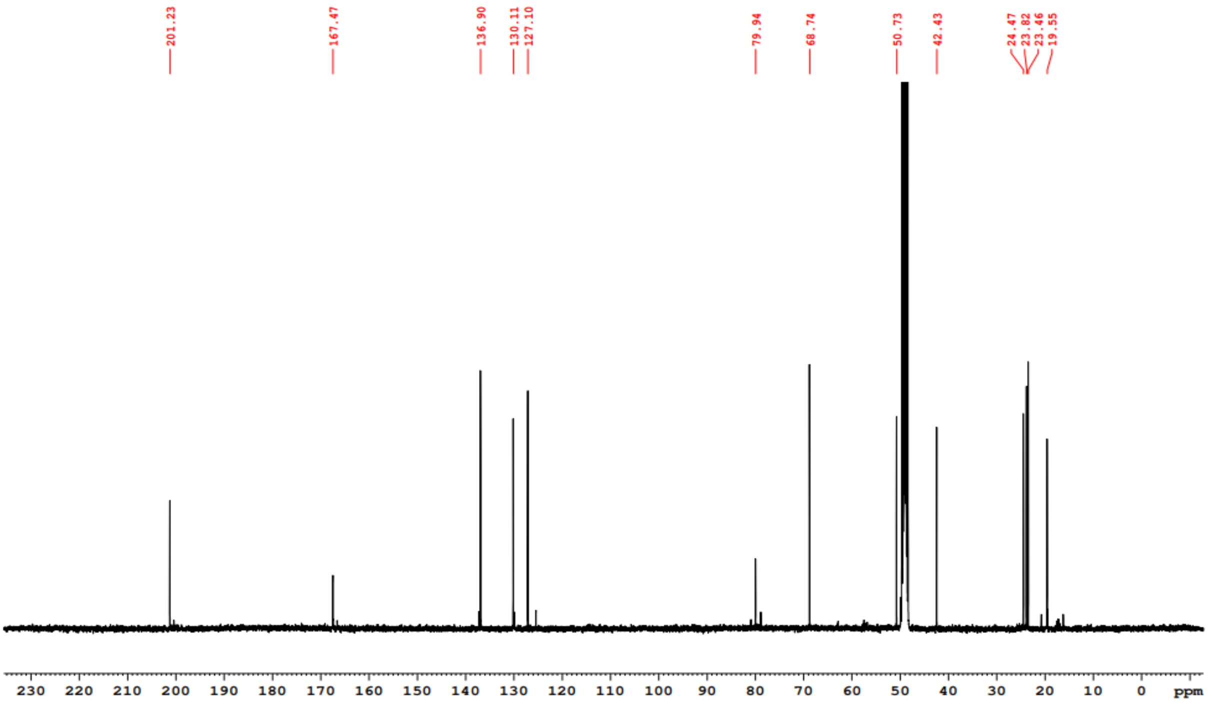

Figure S25. ^{13}C NMR spectrum (100 MHz) of **6** in CD_3OD.

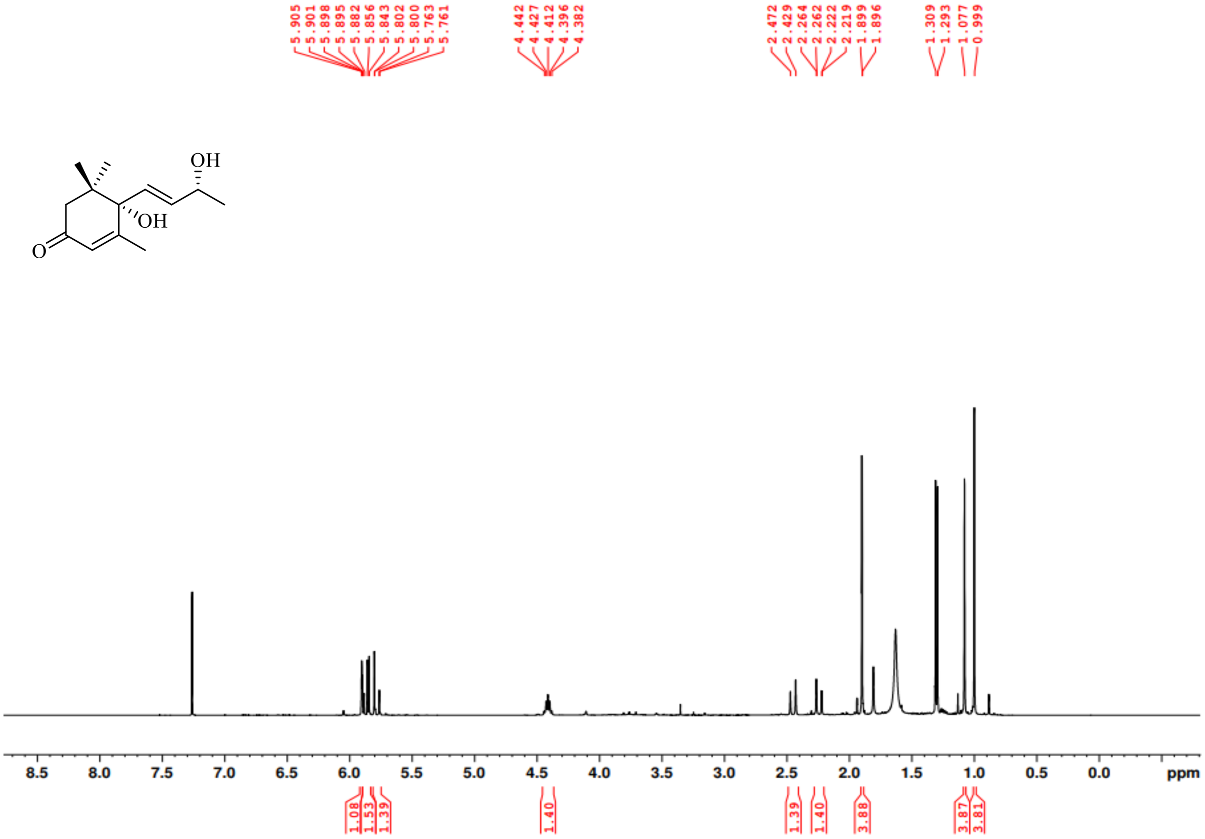

Figure S26. ^{1}H NMR spectrum (400 MHz) of **6** in $CDCl_3$.

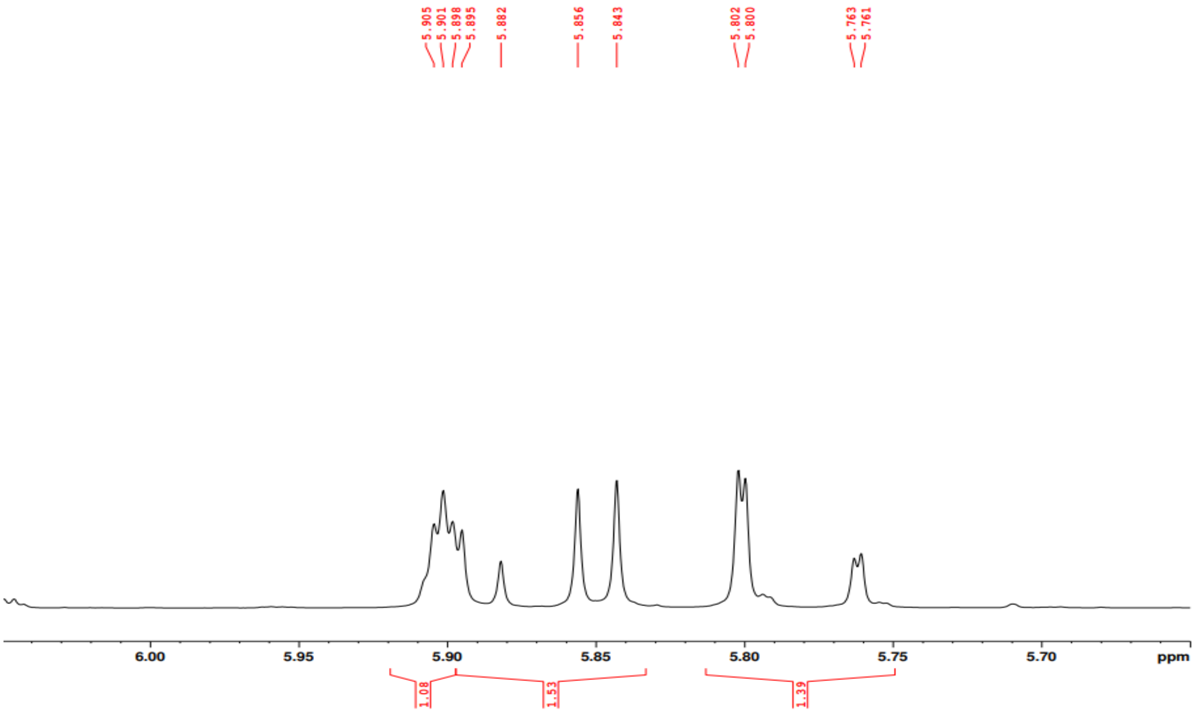

Figure S27. ^{1}H NMR spectrum (400 MHz) (expansion 1) of **6** in $CDCl_3$.

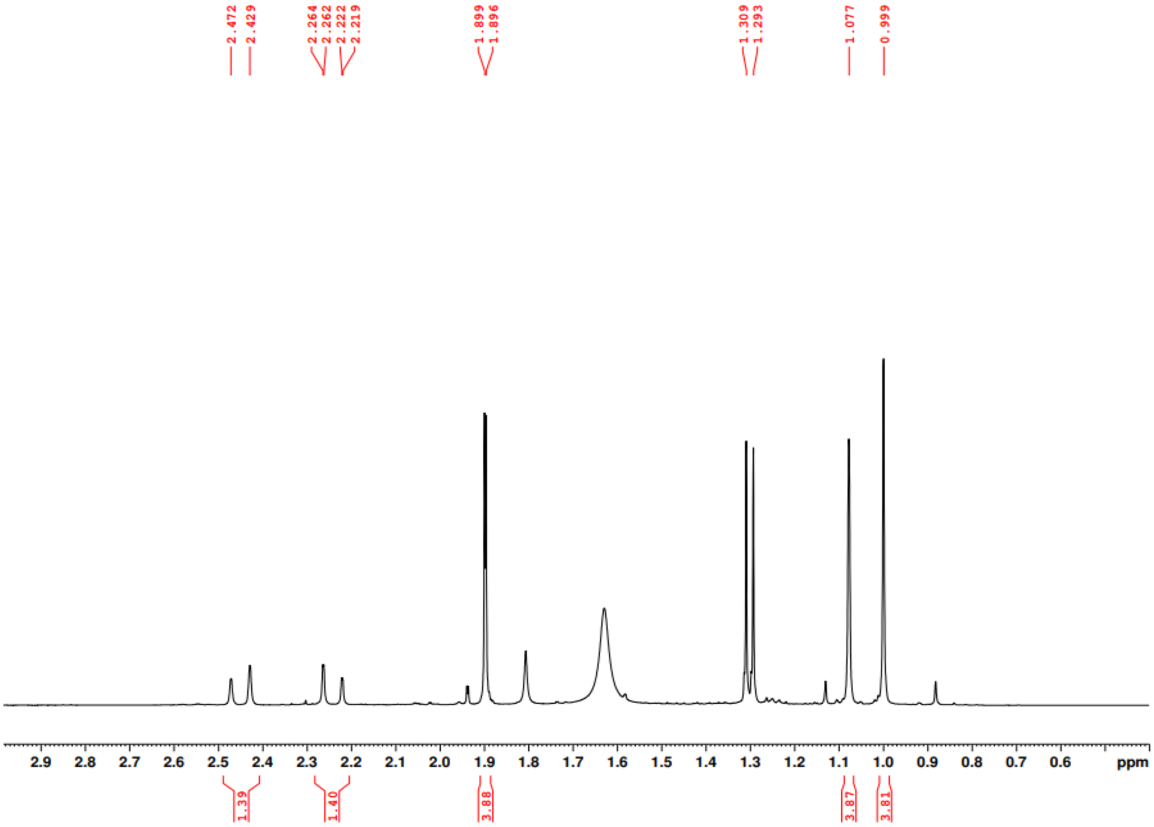

Figure S28. ^{1}H NMR spectrum (400 MHz) (expansion 2) of **6** in $CDCl_3$.

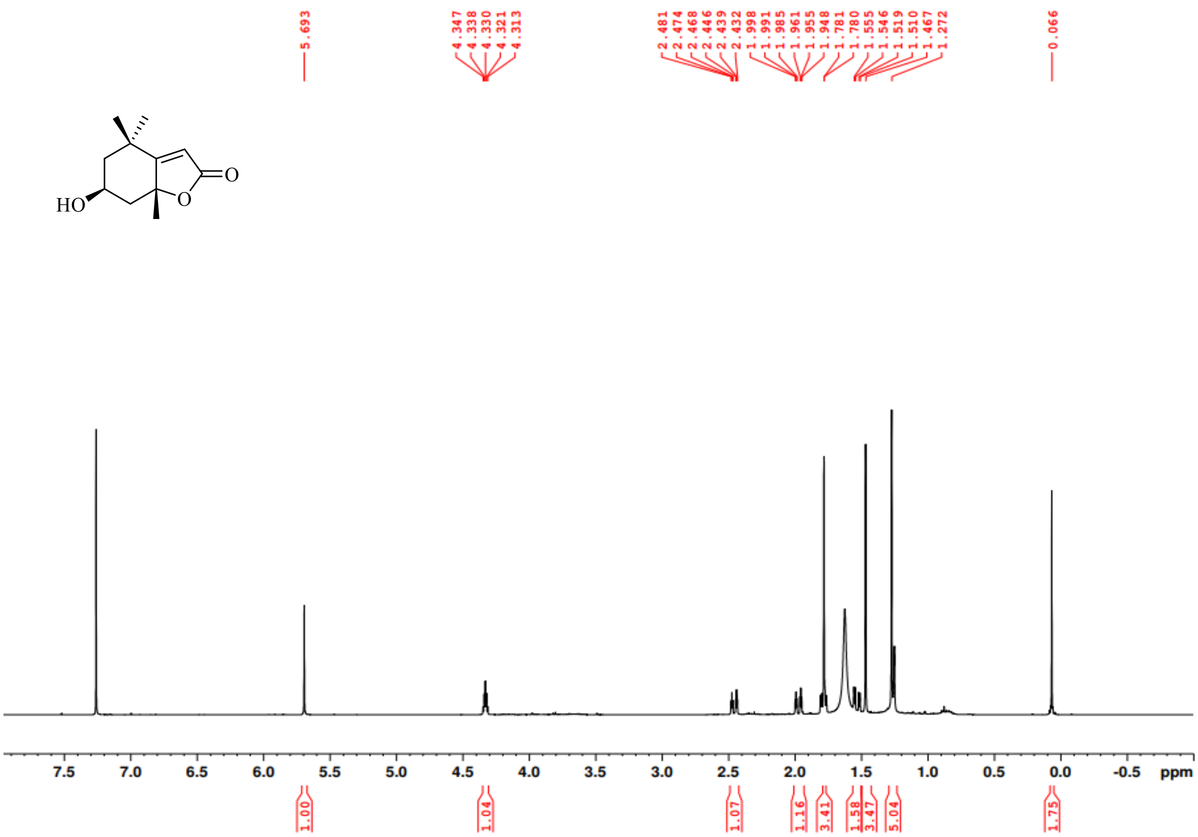

Figure S29. ^{1}H NMR spectrum (400 MHz) of **7** in $CDCl_3$.

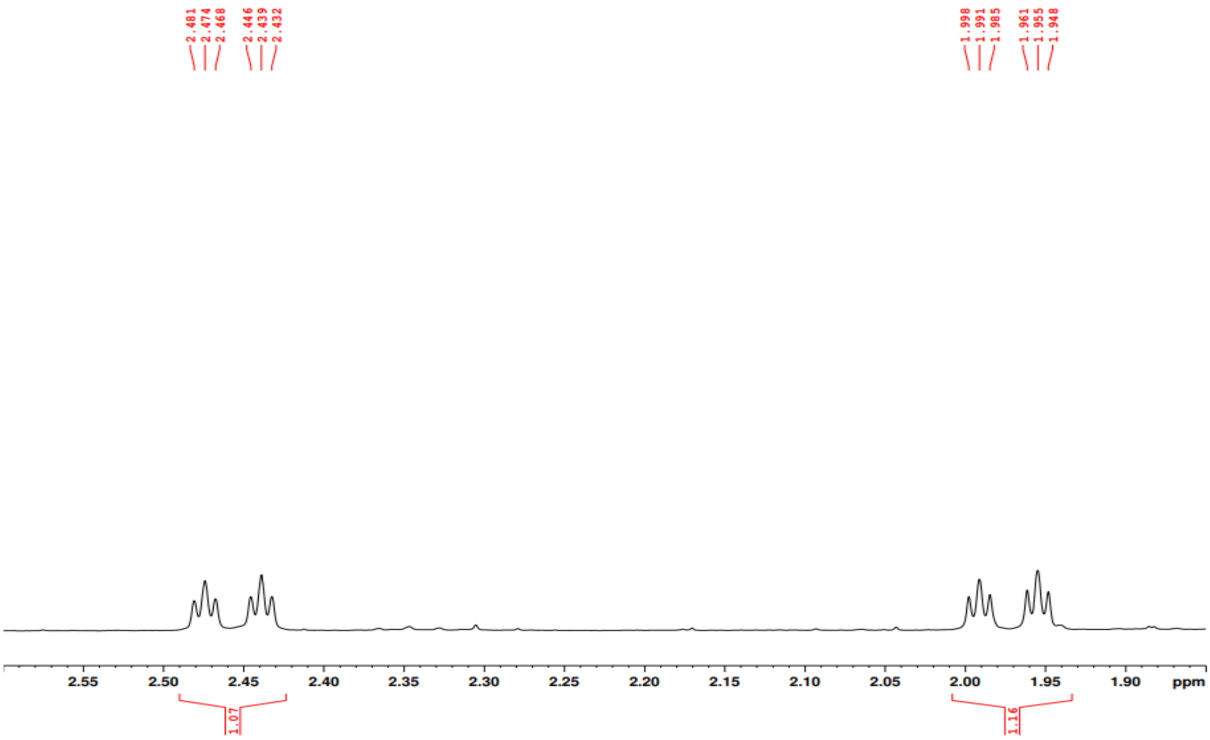

Figure S30. ^{1}H NMR spectrum (400 MHz) (expansion 1) of **7** in $CDCl_3$.

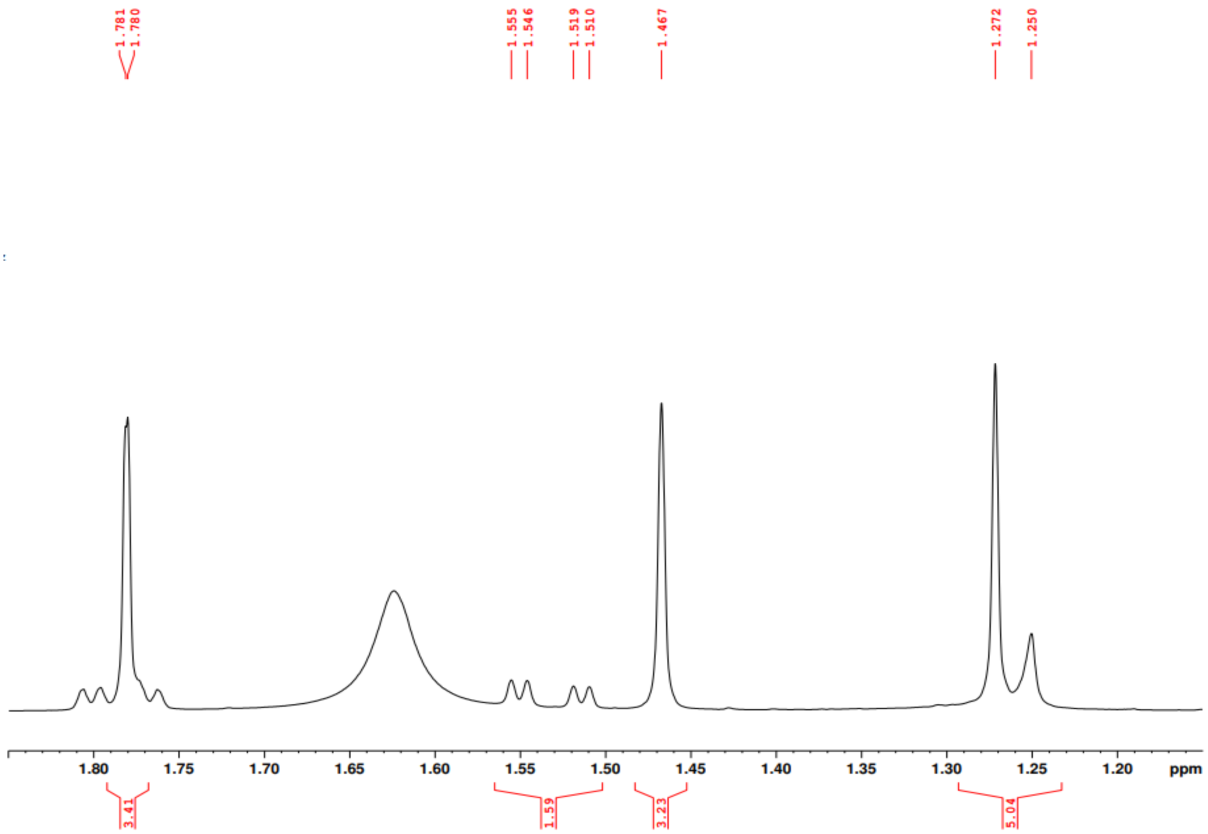

Figure S31. ^{1}H NMR spectrum (400 MHz) (expansion 2) of **7** in $CDCl_3$.

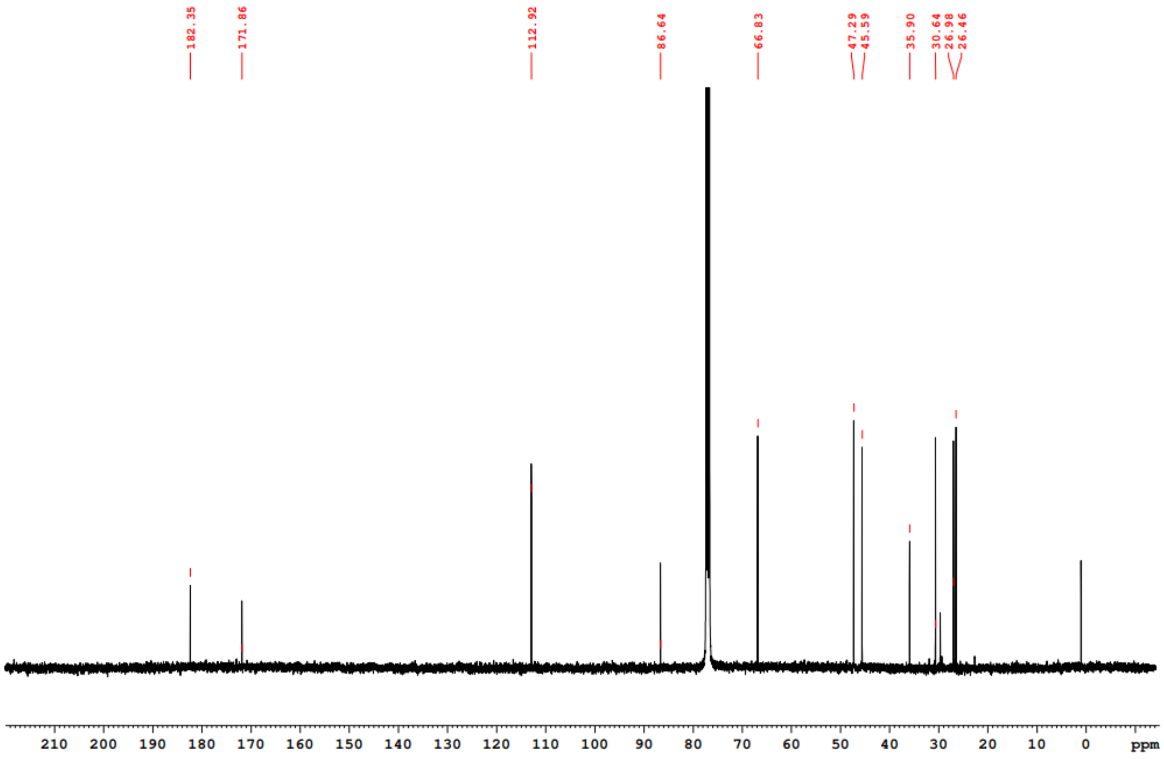

Figure S32. ^{13}C NMR spectrum (100 MHz) of **7** in $CDCl_3$.

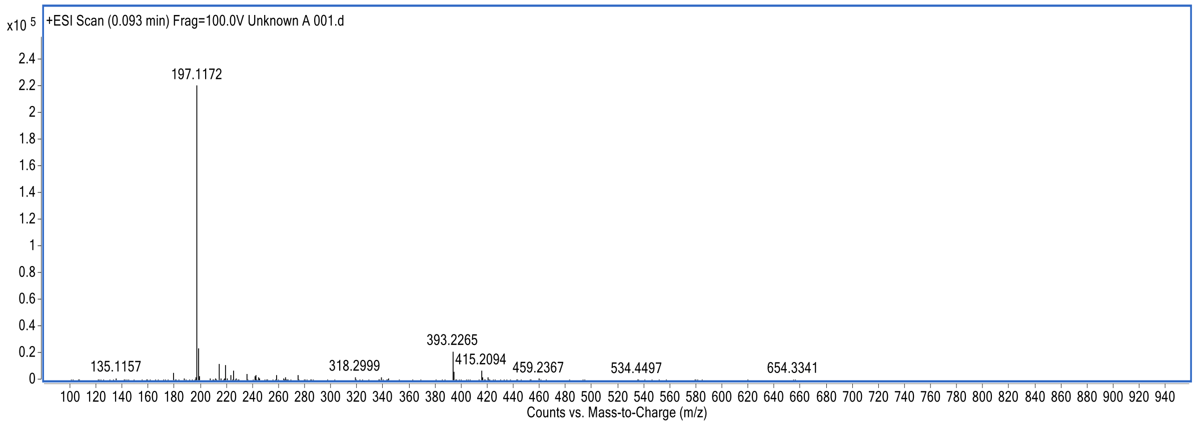

Figure S33. MS chromatogram (positive mode) of **7**.

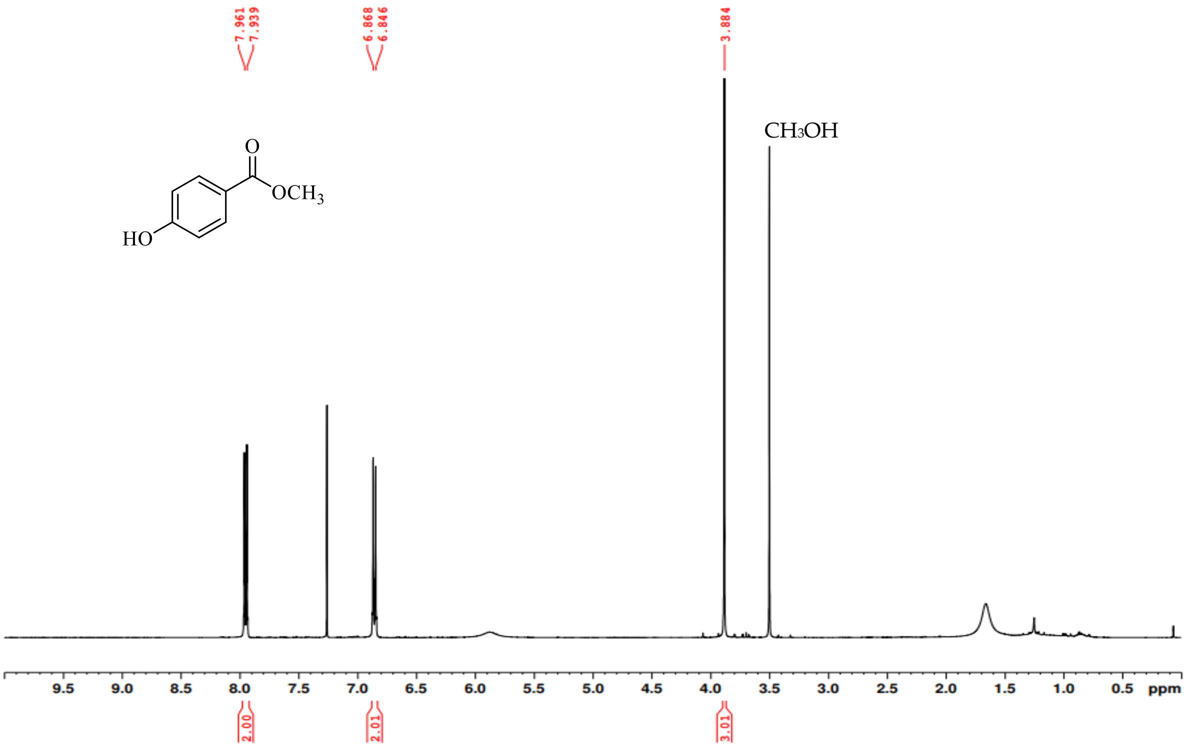

Figure S34. ^{1}H NMR spectrum (400 MHz) of **8** in $CDCl_3$.

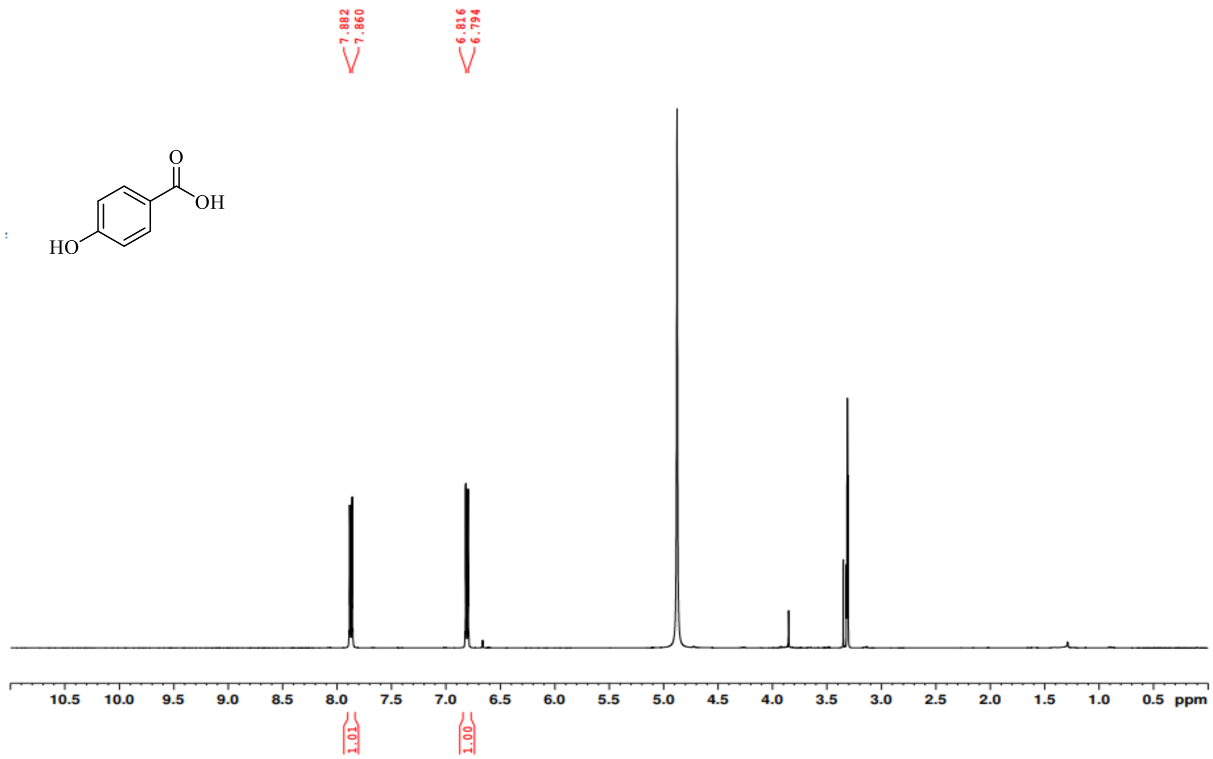

Figure S35. ^{1}H NMR spectrum (400 MHz) of **9** in CD_3OD.

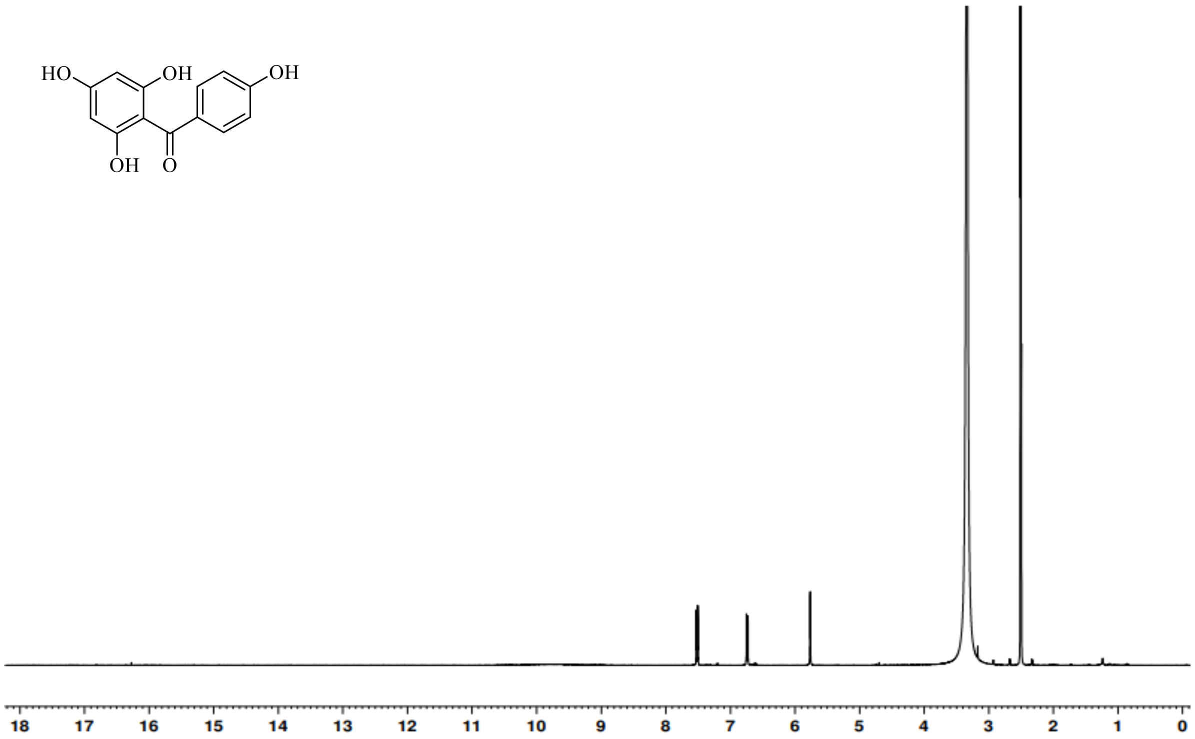

Figure 36. ^{1}H NMR spectrum (400 MHz) of **3a** in DMSO-d_6.

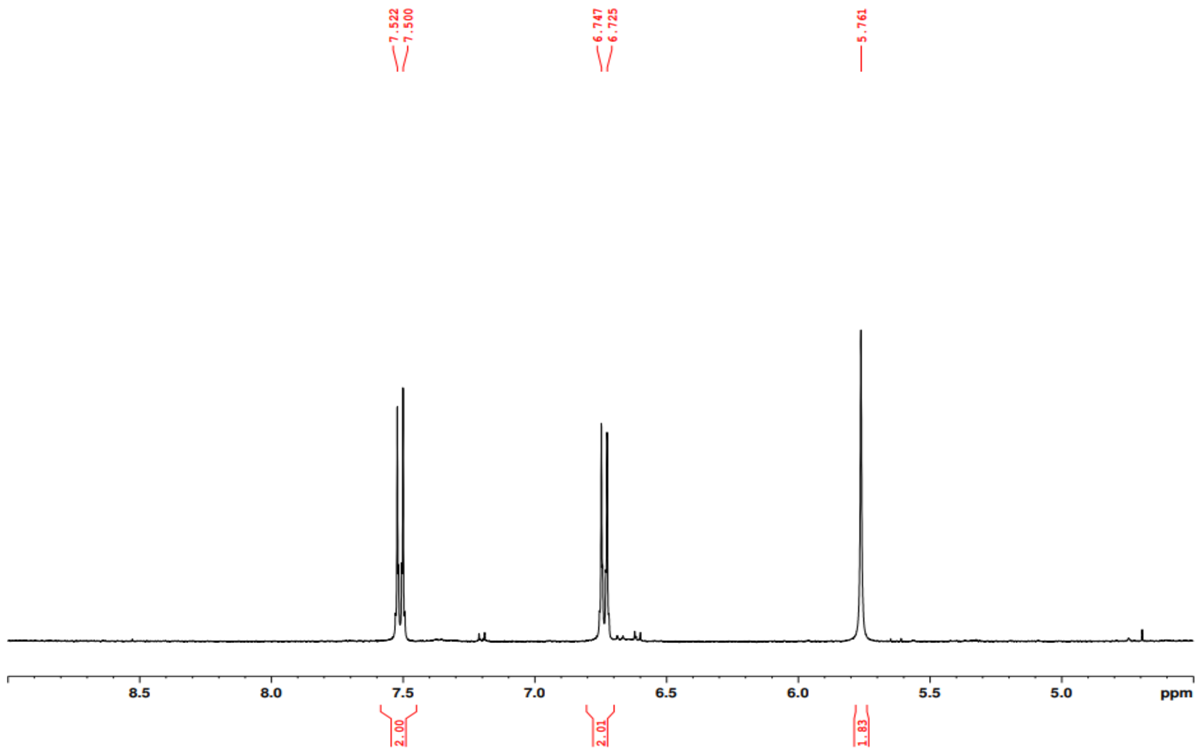

Figure S37. ^{1}H NMR spectrum (400 MHz) (expansion 1) of **3a** in DMSO-d_6.

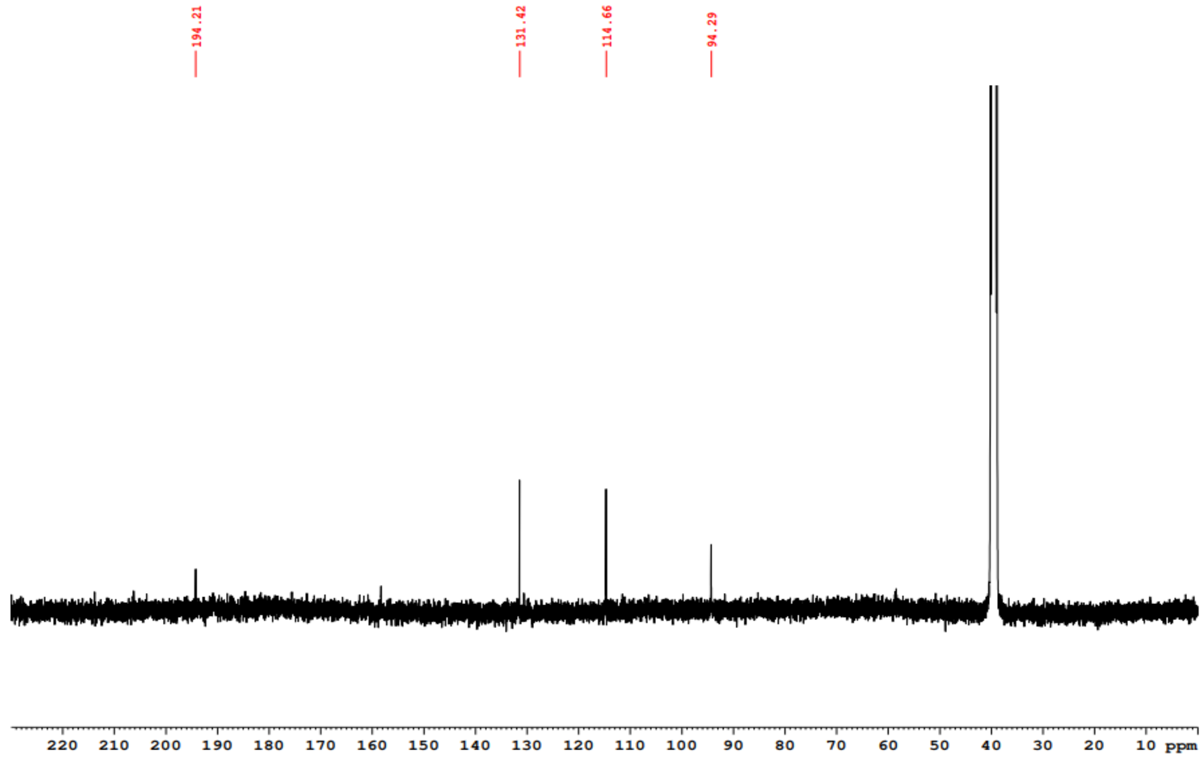

Figure S38. ^{13}C NMR spectrum (100 MHz) of **3a** in DMSO-d_6.

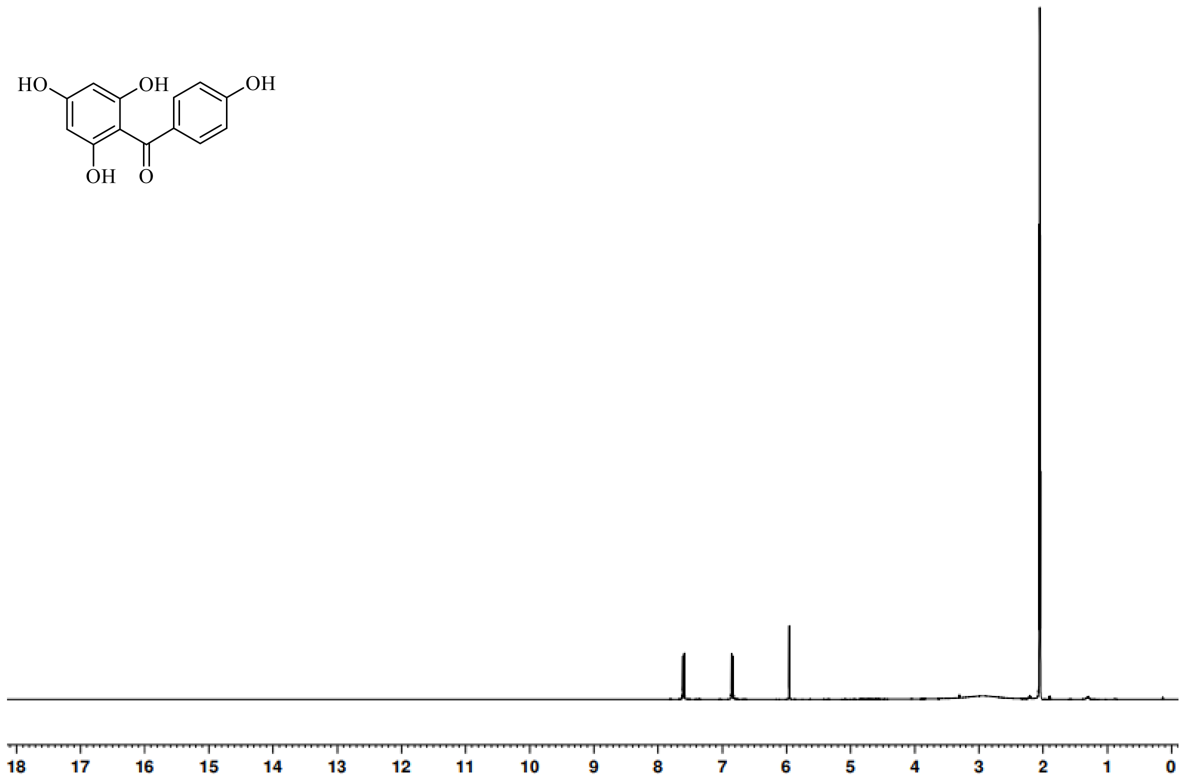

Figure S39. ^{1}H NMR spectrum (400 MHz) of **3a** in acetone-d_6.

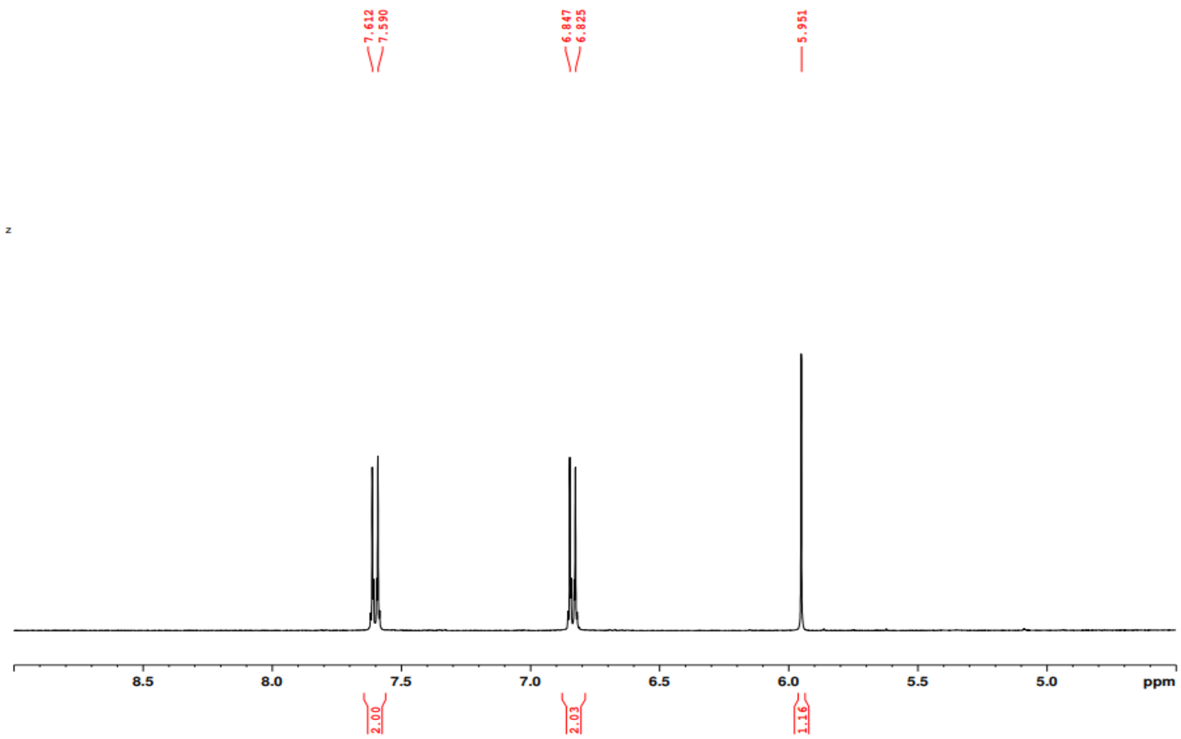

Figure S40. ^{1}H NMR spectrum (400 MHz) (expansion 1) of **3a** in acetone-d_6.

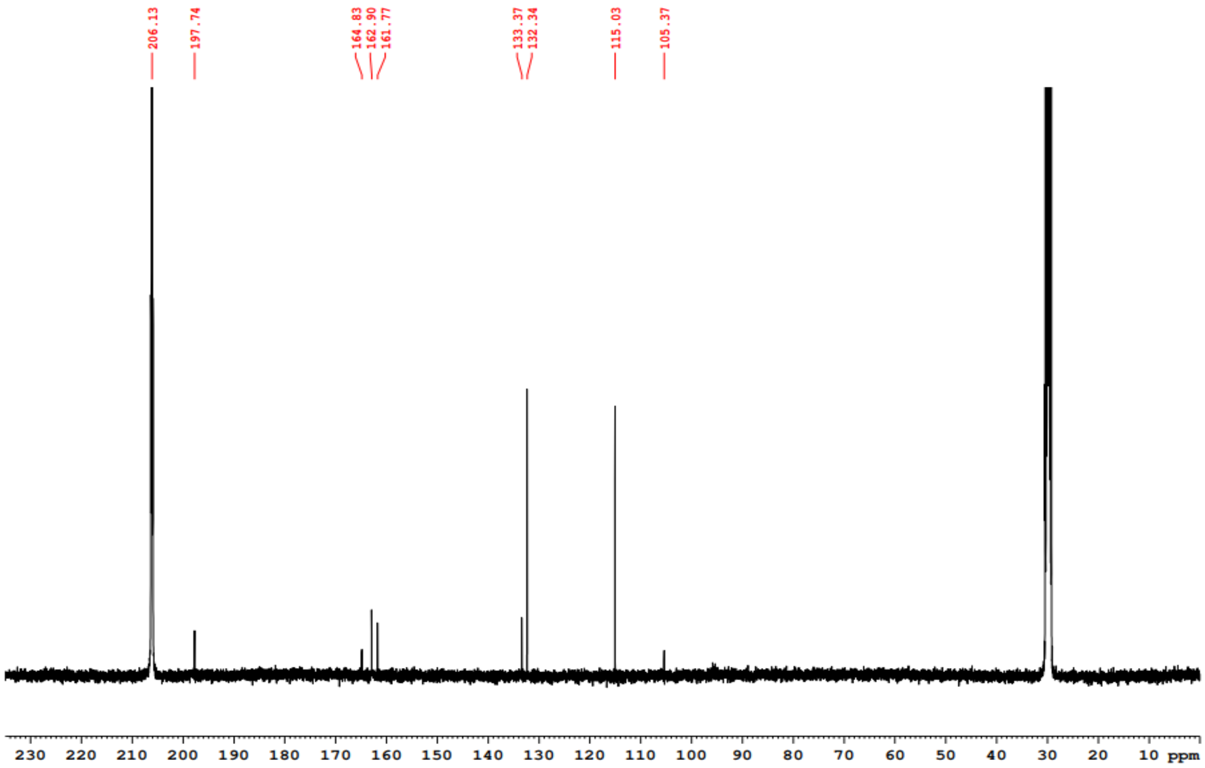

Figure S41. ^{13}C NMR spectrum (100 MHz) of **3a** in acetone-d_6.

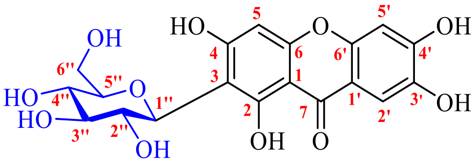

1

Table S1. ^{1}H and ^{13}C NMR spectroscopic data for compound **1** in DMSO-d_6 compared with data reported in the literature.

Position	**1**[a]		[19]		[20]	
	δ_H, multi. (J in Hz)	δ_C	δ_H, multi. (J in Hz)	δ_C	δ_H, multi. (J in Hz)	δ_C
1	-	101.3	-	101.29	-	101.2
2	-	161.8	-	161.76	-	161.7
3	-	107.6	-	107.56	-	107.5
4	-	163.9	-	163.81	-	163.8
5	6.37, s	93.3	6.35, s	93.31	6.40, s	93.3
6	-	156.2	-	156.21	-	156.2
7	-	179.1	-	179.07	-	179.0
1′	-	111.7	-	111.63	-	111.4
2′	7.37, s	108.0	7.36, s	108.00	7.41, s	107.8
3′	-	143.8	-	143.76	-	143.9
4′	-	150.8	-	154.17	-	150.9
5′	6.86, s	102.6	6.84, s	102.56	6.86, s	102.4
6′	-	154.1	-	150.82	-	154.6
1′′	4.59, d (9.8)	73.1	4.58, d (9.8)	73.09	4.60, d (8.3)	73.1
2′′	4.04, t (9.0)	70.2	4.03, dd (9.1, 9.0)	70.62	4.03, t (9.5)	70.3
3′′	3.20-3.10, m	79.0	3.20, m	79.14	3.16, m	79.0
4′′	3.20-3.10, m	70.7	3.14, m	70.26	3.16, m	70.6
5′′	3.20-3.10, m	81.6	3.16, m	81.53	3.16, m	81.5
6′′	3.40, dd, (11.6, 5.8)	61.5	3.40, dd (11.6, 5.5)	62.78	3.40, dd (11.0, 2.1)	61.4
	3.69, d (10.8)	-	3.68, br.d (11.6)	-	3.60, dd (11.0, 4.6)	-
2-OH	13.76, s	-	-	-	13.80, s	-

[a] Calibration of DMSO-d_6 δ_H 2.50/δ_C 39.5 ppm.

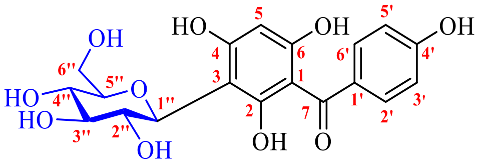

2

Table S2. ^{1}H and ^{13}C NMR spectroscopic data for compound **2** in DMSO-d_6 compared with data reported in the literature.

Position	**2**[a]		[21]	
	δ_H, multi. (*J* in Hz)	δ_C	δ_H, multi. (*J* in Hz)	δ_C
1	-	107.0	-	107.0
2	-	157.4	-	157.4
3	-	103.7	-	103.7
4	-	159.0	-	159.0
5	5.96, s	94.9	5.95, s	94.8
6	-	156.7	-	156.7
7	-	194.7	-	194.6
1′	-	130.7	-	130.7
2′/6′	7.56, d (8.7)	131.5	7.56, d (8.6)	131.5
3′/5′	6.78, d (8.7)	114.6	6.78, d (8.6)	114.6
4′	-	161.4	-	161.4
2-OH	9.35, s	-	9.33, bs	-
4-OH	9.54, s	-	9.52, s	-
6-OH	9.59, s	-	9.57, s	-
4′-OH	10.21, s	-	10.19, s	-
1″	4.59, d (9.8)	74.7	4.59, d (9.6)	74.7
2″	3.65–3.54, m	71.9	3.57, m	71.8
3″	3.23–3.15, m	78.3	3.20, m	78.3
4″	3.23–3.15, m	69.6	3.20, m	69.6
5″	3.23–3.15, m	81.1	3.20, m	81.1
6″	3.65–3.54, m	60.5	3.62, m	60.5
	3.52–3.44, m		3.48, m	-
OH	4.92, d (3.9)	-	4.92, d (3.0)	-
OH	4.88, d (3.8)	-	4.88, d (3.0)	-
OH	4.59, t (5.6)	-	4.59, t (5.7)	-
OH	4.77, d (5.2)	-	-	-

[a] Calibration of DMSO-d_6 δ_H 2.50/δ_C 39.5 ppm.

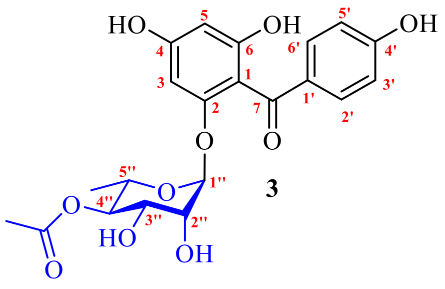

3

Table S3. ^{1}H and ^{13}C NMR spectroscopic data for compound **3** in CD_3OD compared with data reported in the literature, and for compound **3** in DMSO-d_6.

Position	**3**[a] (in CD_3OD)		[22] (in CD_3OD)		**3** (in DMSO-d_6)	
	δ_H, multi. (J in Hz)	δ_C	δ_H, multi. (J in Hz)	δ_C	δ_H, multi. (J in Hz)	δ_C
1	-	109.6	-	109.7	-	109.4
2	-	157.8	-	158.0	-	154.8
3	6.24, d (2.1)	94.9	6.25, d (2.0)	95.1	6.10, d (1.9)	93.4
4	-	162.9	-	163.1	-	159.4
5	6.08, d (2.1)	98.0	6.08, d (2.0)	98.1	6.04, d (1.9)	96.7
6	-	160.4	-	160.6	-	156.4
7	-	197.6	-	197.7	-	192.8
1′	-	132.9	-	133.1	-	130.1
2′/6′	7.62, d (8.8)	132.7	7.6, d (8.7)	132.8	7.55, d (8.8)	131.3
3′/5′	6.84, d (8.8)	116.2	6.8, d (8.7)	116.3	6.80, d (8.8)	115.1
4′	-	163.4	-	163.1	-	161.8
4-OH	-	-	-	-	9.65, s	-
6-OH	-	-	-	-	9.57, s	-
4′-OH	-	-	-	-	10.26, s	-
1′′	5.29, d (1.7)	99.6	5.29, d (1.4)	99.8	5.21, d (1.5)	97.8
2′′	3.52, m	71.5	3.51, m	71.6	3.46–3.41, m	69.8
3′′	3.10, dd (9.8, 3.4)	69.7	3.09, m	69.9	3.23–3.15, m	67.5
4′′	4.80, t (9.8)	75.0	3.79, m	79.2	4.67, t (9.8)	73.3
5′′	3.47, m	68.7	3.48, m	68.8	3.36 (overlap)*	66.9
2′′-OH	-	-	-	-	5.18, d (4.4)	-
3′′-OH	-	-	-	-	4.77, d (6.4)	-
4′′-OCO<u>C</u>H3	2.06, s	21.0	2.0, s	21.1	1.98, s	20.9
4′′-O<u>C</u>OCH3	-	172.6	-	172.7	-	170.0
5′′-CH_3	1.05, d (6.3)	17.8	1.05, d (6.0)	17.9	0.90, d (6.2)	17.4

[a] Calibration of CD_3OD δ_H 3.31/δ_C 49.0 ppm, and DMSO-d_6 δ_H 2.50/δ_C 39.5 ppm.

* Overlapped with the solvent peak.

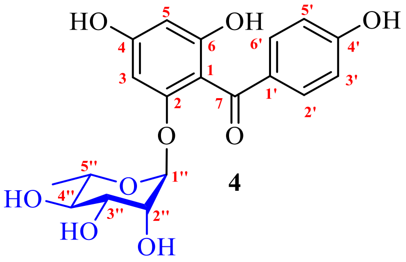

4

Table S4. ^{1}H NMR spectroscopic data for compound **4** in CD_3OD compared with data reported in the literature.

Position	**4**[a]	[23]	[24]
	δ_H, multi. (J in Hz)	δ_H, multi. (J in Hz)	δ_H, multi. (J in Hz)
1	-	-	-
2	-	-	-
3	6.27, d (2.1)	6.34, d (2.0)	6.29, d (2.0)
4	-	-	-
5	6.04, d (2.1)	6.11, d (2.0)	6.07, d (2.0)
6	-	-	-
7	-	-	-
1′	-	-	-
2′/6′	7.58, d (8.7)	7.65, d (8.5)	7.61, d (8.5)
3′/5′	6.78, d (8.7)	6.85, d (8.5)	6.81, d (8.5)
4′	-	-	-
1′′	5.19, d (1.6)	5.26, d (1.0)	5.22, d (1.0)
2′′	3.39–3.35, m	3.50–3.45, m	3.43, m
3′′	3.09, dd (9.5, 3.4)	3.16, dd (9.5, 3.0)	3.12, dd (9.5, 3.5)
4′′	3.29–3.21, m	3.32, dd (9.5, 3.0)	3.28, dd (9.5, 3.5)
5′′	3.45–3.39, m	3.50–3.45, m	3.40, m
5′′-CH_3	1.16, d (6.2)	1.23, d (6.0)	1.19, d (6.0)

[a] Calibration of CD_3OD δ_H 3.31 ppm.

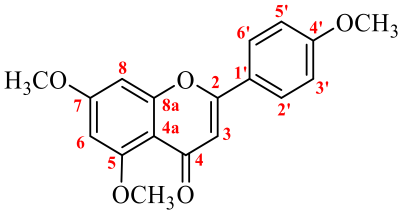

5

Table S5. ^{1}H and ^{13}C NMR spectroscopic data for compound **5** in $CDCl_3$ compared with data reported in the literature.

Position	**5**[a]		[25]	
	δ_H, multi. (J in Hz)	δ_C	δ_H, multi. (J in Hz)	δ_C
2	-	164.0	-	162.1
3	6.58, s	104.3	6.62, s	107.7
4	-	182.5	-	177.6
4a	-	105.5	-	109.2
5	-	157.7	-	160.9
6	6.49, d (2.3)	92.6	6.56, d (2.4)	92.8
7	-	165.4	-	163.9
8	6.37, d (2.3)	98.0	6.38, d (2.4)	96.1
8a	-	162.2	-	160.7
1′	-	123.6	-	123.9
2′/6′	7.85, d (9.0)	128.0	7.84, d (8.8)	127.6
3′/5′	7.02, d (9.0)	114.5	7.02, d (8.8)	114.4
4′	-	162.6	-	159.8
5-OCH_3	3.49, s	50.9	3.96, s	56.4
7-OCH_3	3.88, s	55.5	3.92, s	55.7
4′-OCH_3	3.90, s	55.8	3.89, s	55.5

[a] Calibration of $CDCl_3$ δ_H 7.26/δ_C 77.0 ppm.

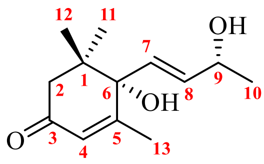

6

Table S6. ^{1}H and ^{13}C NMR spectroscopic data for compound **6** in CD_3OD compared with data reported in the literature.

Position	**6**[a]		[26]		[27]	
	δ_H, multi. (J in Hz)	δ_C	δ_H, multi. (J in Hz)	δ_C	δ_H, multi. (J in Hz)	δ_C
1	-	42.4	-	42.42	-	42.4
2	2.16, d (17.0)	50.7	2.18, d (17.0)	50.73	2.18, d (17.0)	50.7
	2.52, d (17.0)	50.7	2.53, d (17.0)	50.73	2.53, d (17.0)	50.7
3	-	201.2	-	201.27	-	201.2
4	5.88, t (1.2)	127.1	5.90, t (1.5)	127.10	5.89, m	127.1
5	-	167.5	-	167.48	-	167.4
6	-	79.9	-	79.95	-	79.9
7	5.79 (overlap)*	130.1	5.81 (overlap)*	130.10	5.80, m	130.1
8	5.79 (overlap)*	136.9	5.82 (overlap)*	136.92	5.83, m	136.9
9	4.34, m	68.7	4.34, dd (6.5, 4.5)	68.72	4.34, m	68.7
10	1.24, d (6.5)	23.8	1.26, d, (6.5)	23.82	1.26, d (6.5)	23.8
11	1.04, s	23.5	1.06, s	23.46	1.04, s	23.4
12	1.01, s	24.5	1.03, s	24.47	1.06, s	24.5
13	1.92, d (1.3)	19.6	1.94, d (1.5)	19.56	1.94, s	19.5

[a] Calibration of CD_3OD δ_H 3.31/δ_C 49.0 ppm.

* Overlapped signal.

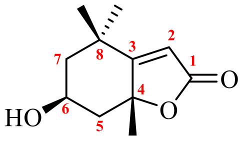

7

Table S7. ^{1}H and ^{13}C NMR spectroscopic data for compound **7** in $CDCl_3$ compared with data reported in the literature.

Position	**7**[a]		[29] Loliolide		*epi*-Loliolide	
	δ_H, multi. (*J* in Hz)	δ_C	δ_H, multi. (*J* in Hz)	δ_C	δ_H, multi. (*J* in Hz)	δ_C
1	-	171.9	-	172.2	-	171.9
2	5.69, s	112.9	5.69, s	112.8	5.68, s	113.0
3	-	182.3	-	182.8	-	181.3
4	-	86.6	-	87.0	-	88.6
5	2.46, ddd (14.1, 2.8, 2.8)	45.6	2.48, ddd (14.0, 2.5, 2.5)	45.6	2.51, ddd (12.8, 4.3, 2.3)	49.7
	1.78, dd (14.4, 2.8)		1.77, dd (14.0, 4.0)		1.31, dd (12.2, 12.2)	
6	4.33, m	66.6	4.34, m	66.7	4.10, m	64.8
7	1.98, ddd (14.1, 2.8, 2.8)	47.3	2.00, ddd (14.5, 2.7, 2.7)	47.2	2.02, ddd (12.8, 4.3, 2.3)	47.8
	1.53, dd (14.4, 3.6)		1.53, dd (14.5, 3.7)		1.31, dd (12.2, 12.2)	
8	-	35.9	-	36.0	-	35.1
4-CH_3	1.78, s	27.0	1.79, s	27.0	1.56, s	25.5
8-CH_3	1.47, s	30.6	1.48, s	30.7	1.29, s	29.9
8-CH_3	1.27, s	26.5	1.27, s	26.5	1.24, s	25.0

[a] Calibration of $CDCl_3$ δ_H 7.26/δ_C 77.0 ppm.

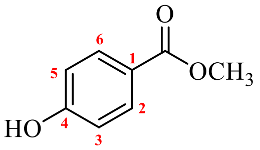

8

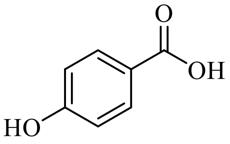

9

Table S8. ^{1}H NMR spectroscopic data for compounds **8** and **9** compared with data reported in the literature.

Position	**8**[a] (in $CDCl_3$)	[30]	**9**[b] (in CD_3OD)	[31]
	δ_H, multi. (J in Hz)	δ_H, multi. (J in Hz)	δ_H, multi. (J in Hz)	δ_H, multi. (J in Hz)
1	-	-	-	-
2/6	7.95, d (8.8)	7.96	7.87, d (8.8)	7.87, d (8.2)
3/5	6.86, d (8.8)	6.89	6.81, d (8.8)	6.80, d (8.2)
4	-	-	-	-
1-$COCH_3$	3.88, s	3.90	-	-
OH	5.88, brs	6.40, brs	-	-

[a] Calibration of $CDCl_3$ δ_H 7.26/δ_C 77.0 ppm.
[b] Calibration of CD_3OD δ_H 3.31/δ_C 49.0 ppm.

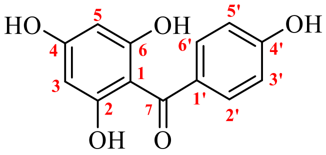

3a

Table S9. ^{1}H and ^{13}C NMR spectroscopic data for compound **3a** in in acetone-d_6 compared with data reported in the literature, and for compound **3a** in DMSO-d_6.

Position	**3a**[a] (acetone-d_6)		[50] (in acetone-d_6)		**3a**[b] (DMSO-d_6)	
	δ_H, multi. (J in Hz)	δ_C	δ_H, multi. (J in Hz)	δ_C	δ_H, multi. (J in Hz)	δ_C
1	-	105.4	-	106.6	-	99.8
2	-	162.9	-	164.0	-	158.3
3	5.95, s	95.8	5.99, s	96.9	5.76, s	94.3
4	-	164.8	-	164.6	-	158.3
5	5.95, s	95.8	5.99, s	96.9	5.76, s	94.3
6	-	162.9	-	164.0	-	158.3
7	-	197.7	-	198.9	-	194.2
1′	-	133.4	-	134.4	-	130.6
2′/6′	7.60, d, (8.8)	132.3	7.62, d (8.7)	133.4	7.51, d (8.7)	131.4
3′/5′	6.84, d, (8.8)	115.0	6.86, d (8.7)	116.1	6.74, d (8.7)	114.7
4′	-	161.8	-	162.8	-	158.3
2-OH	-	-	10.22, brs.	-	-	-
4-OH	-	-	9.14, brs.	-	-	-
6-OH	-	-	10.22, brs.	-	-	-
4′-OH	-	-	9.14, brs.	-	-	-

[a] Calibration of acetone-d_6 δ_H 2.05/δ_C 29.8 ppm.
[b] Calibration of DMSO-d_6 δ_H 2.50/δ_C 39.5 ppm.

Table S10. Lipinski rule of 5 for **3a** and **5**.

Compound	MW<br>< 500	LogP<br>< 5	HBD<br>< 5	HBA<br>< 10	Rule's satisfied
**3a**	246.2	1.74	4	5	5
**5**	312.3	3.49	0	5	5

Table S11. Predicted ADMET properties of **3a** and **5**.

Compound	Adsorption		Distribution		Metabolism				Excretion		Toxicity	
	Water solubility	Intestinal absorption (human)	BBB permeability	CNS permeability	CYP				Renal OCT2 substrate	Total Clearance	AMES toxicity	Hepatotoxicity
					2D6	3A4	2D6	3A4				
					Substate		Inhibitor					
	Numeric (log mol/L)	Numeric (% Absorbed)	Numeric (log BB)	Numeric (log PS)	Categorical (Yes/No)				Categorical (Yes/No)	Numeric (log ml/min/kg)	Categorical (Yes/No)	
**3a**	-3.09	61	-0.954	-2.52	No	No	No	Yes	No	0.495	No	No
**5**	-4.352	98	0.257	-2.12	No	Yes	No	No	Yes	0.819	No	No

Table S12. Preliminary screening results for the DPPH radical scavenging and α-glucosidase inhibitory activities of compounds **1**–**5** and **3a**.

Compound	DPPH Scavenging Activity (%) at 50 μg/mL			Average	α-Glucosidase Inhibitory Activity (%) at 50 μg/mL			Average
**1**	84.23	79.02	89.67	84.31	-8.00*	-8.54*	-7.41*	-8.00*
**2**	18.37	17.20	19.55	18.37	4.29	4.12	4.58	4.33
**3**	2.23	2.05	2.41	2.23	4.29	4.67	3.90	4.29
**4**	2.23	2.43	2.01	2.22	10.06	9.25	10.84	10.08
**5**	0.56	0.60	0.51	0.56	46.87	50.27	43.58	46.90
**3a**	15.58	14.48	16.91	15.56	54.28	51.26	58.11	54.55

*The absorbance of the sample was higher than that of the control, resulting in negative inhibition (%) values. This observation may be attributed to the poor solubility of the sample in the assay buffer, which could have caused increased turbidity or optical interference, leading to artificially elevated absorbance readings.
